# LC-HRMS Profiling and Antidiabetic, Antioxidant, and Antibacterial Activities of *Acacia catechu* (L.f.) Willd

**DOI:** 10.1155/2021/7588711

**Published:** 2021-08-13

**Authors:** Babita Aryal, Bikash Adhikari, Niraj Aryal, Bibek Raj Bhattarai, Karan Khadayat, Niranjan Parajuli

**Affiliations:** ^1^Biological Chemistry Lab, Central Department of Chemistry, Tribhuvan University, Kirtipur, Kathmandu, Nepal; ^2^Pharmaceutical Institute, Department of Pharmaceutical Biology, University of Tübingen, Germany

## Abstract

*Acacia catechu* (L.f.) Willd is a profoundly used traditional medicinal plant in Asia. Previous studies conducted in this plant are more confined to extract level. Even though bioassay-based studies indicated the true therapeutic potential of this plant, compound annotation was not performed extensively. This research is aimed at assessing the bioactivity of different solvent extracts of the plant followed by annotation of its phytoconstituents. Liquid chromatography equipped with high resolution mass spectrometry (LC-HRMS) is deployed for the identification of secondary metabolites in various crude extracts. On activity level, its ethanolic extract showed the highest inhibition towards *α-*amylase and *α*-glucosidase with an IC_50_ of 67.8 ± 1 *μ*g/mL and 10.3 ± 0.1 *μ*g/mL respectively, inspected through the substrate-based method. On the other hand, the plant extract showed an antioxidant activity of 23.76 ± 1.57 *μ*g/mL, measured through radical scavenging activity. Similarly, ethyl acetate and aqueous extracts of *A. catechu* showed significant inhibition against *Staphylococcus aureus* with a zone of inhibition (ZoI) of 13 and 14 mm, respectively. With the LC-HRMS-based dereplication strategy, we have identified 28 secondary metabolites belonging to flavonoid and phenolic categories. Identification of these metabolites from *A. catechu* and its biological implication also support the community-based usage of this plant and its medicinal value.

## 1. Introduction

*Acacia catechu* (L.f.) Willd is a deciduous and gregarious tree with a light feathery crown native to Nepal, India, and Myanmar and one of the most promising medicinal plants of the family Fabaceae [1]. *A. catechu* has received attention as a potential source of bioactive secondary metabolites to be used for the formulation of pharmaceutical products [2, 3]. Various parts of plant extracts were known to have strong antioxidant [2], antimicrobial [4], anti-inflammatory [5], antihyperglycemic [6], and immunomodulatory [7] activities. Although several secondary metabolites have been identified from *A. catechu*, the molecules, catechin, epicatechin, and quercetin, are the principal contributor to therapeutical properties [8]. Nowadays, plant-based secondary metabolites are extensively used in the management of various infectious diseases and achieved clinical benefits in the health care system.

Diabetes mellitus (DM) is a leading cause of hyperglycemia, and carbohydrate-hydrolase inhibitors, such as inhibitors for *α*-amylase and *α*-glucosidase, offer an effective strategy to lower the level of postprandial hyperglycemia via control of dietary carbohydrates and glycogen breakdown [9]. Moreover, microbial infections have undermined the existing antibiotic-based treatment era and raised the mortality rate in patients with higher medical expenses and extended hospital stays [10]. Around the world, about 28,000 plant taxa have been known for their medicinal values and about 3000 plant species possess ethnopharmacological uses for the management of DM and others [11]. Different explorers had effectively shown the inhibitory abilities of natural products towards digestive enzymes, hence reducing hyperglycemia [12–14].

Hyperglycemia stimulates the autooxidation of glucose to generate free radicals [15]. Free radicals are created constantly in the body during metabolism as they are required to serve various essential functions essential for survival [16]. A body with a weak defense system is unable to counteract these increased radicals, which ultimately leads to imbalance, and this condition of more free radicals than antioxidants is known as oxidative stress. Excess free radicals and reactive oxygen species, beyond the scavenging capacities of the cellular antioxidant system, are involved in several human diseases and complications like arteriosclerosis, cancer, the aging process, diabetes, cardiovascular disease, nerve damage, blindness, and nephropathy [15, 17–20]. Antioxidants from natural products especially fruits, vegetables, herbs, and spices are effective in reducing diabetic vascular complications [15] and have diverse pharmaceutical properties [21, 22].

Plant products have played the important role in the development of new therapeutics with milder adverse side effects than commercial drugs [23]. Consequently, it is important to recognize and measure all the secondary metabolites to ensure the biological research reliability and reproducibility over the pharmacological benefits and/or hazards. Currently, liquid chromatography with high resolution mass spectrometry (LC-HRMS) has emerged as a leading tool for detecting and identifying pharmacologically active secondary metabolites [24, 25]. Nuclear magnetic resonance spectroscopy and mass spectrometry followed by chemometric tools are the most used analytical methods of annotation [26]. Additionally, LC-HRMS is useful for exact mass measurement as well as for molecular formula generation of any unknown molecules, parent ions, and fragment ions in the plant extracts [27]. LC-HRMS not only allows for the determination of chemical structure but also offers excellent sensitivity to the low amount of sample in a short time and also plays an important role in the screening of flavonoids and other phenolic contents [28, 29]. Thus, this study was carried out for method validation for some biochemical assays and molecular profiling in the ethanolic extract of *A. catechu*.

## 2. Materials and Methods

### 2.1. Chemicals

The *α*-glucosidase from *Saccharomyces cerevisiae* (CAS No. 9001-42-7), porcine pancreas *α*-amylase (CAS No. 9000-90-2), 4-nitrophenyl-*α*-D-glucopyranoside (pNPG) (CAS No. 3767-28-0), 2-chloro-4-nitrophenyl-*α*-D-maltotrioside (CNPG3) (CAS No.118291-90-0), acarbose (CAS No. 56180-94-0), and quercetin (CAS No. 117-39-5) were purchased from Sigma-Aldrich, Germany. Gallic acid, 2,2-diphenyl-1-picrylhydrazyl (DPPH) radical, Folin-Coicalteu reagent, resazurin sodium, and other chemicals and solvents were purchased from Molychem, Himedia, and Fisher Scientific, India.

### 2.2. Collection and Identification of the Plant

*A. catechu* was collected from Syangja District of Gandaki Province with coordinates 27° 58′ N, 83° 46′ E Nepal, in January 2020. Its taxonomy was verified in voucher specimen TUCH-201011 by the Central Department of Botany, Tribhuvan University, Kirtipur, Nepal. Its major phytoconstituents and ethnopharmacological application are mentioned in Table S1.

### 2.3. Preparation of Crude Extracts and Fractionation

The barks of *A. catechu* were shade dried at room temperature and pulverized. The powder was soaked in ethanol for 24 hours and filtered. The same process was repeated for three successive days, and collected filtrate was concentrated in a vacuum in a rotary evaporator below 45°C. Fractionation was carried out by dissolving 50 g of crude ethanolic extract into distilled water and then partitioned three times with hexane, dichloromethane (DCM), and ethyl acetate successively to obtain respective solvent-solvent fractions.

### 2.4. Total Phenolic Content

The Folin-Coicalteu method was employed for the estimation of total phenolic content (TPC) as described by Lu *et al*. [30] with slight modifications. 20 *μ*L of sample (0.5 mg/mL) plant extracts/standard followed by 100 *μ*L of Folin-Coicalteu reagent (1 : 10; *v*/*v*) was added in a 96-well microtiter plate, and initial absorbance was taken. Then, 80 *μ*L of 1 M Na_2_CO_3_ solution was added to the above mixture making a final volume of 200 *μ*L and incubated for 25 minutes. Absorbance was measured at 765 nm by using Synergy LX Multi-Mode Reader with Gen5 3.08.01 software. The TPC was determined using a calibration curve with gallic acid, and results were expressed as milligrams of gallic acid equivalent per gram of dry weight of the extract (mg GAE/g). Triplicates of each measurement were carried out for validation.

### 2.5. Total Flavonoid Content

Total flavonoid content (TFC) were estimated by the AlCl_3_ method, based on the formation of a complex between AlCl_3_ and flavonoid with a maximum absorbance at 415 nm [31]. Briefly, 20 *μ*L of each extract (0.5 mg/mL) was separately mixed with 60 *μ*L ethanol and 5 *μ*L 10% AlCl_3_. Subsequently, 5 *μ*L of 1 M potassium acetate and 110 *μ*L distilled water were supplemented into each well, and the reaction mixture was allowed to stand for 25 minutes. Triplicates of each measurement were carried out to verify experimental reproducibility. The TFC was determined using a calibration curve with quercetin, and results were expressed as milligrams of quercetin equivalent per gram dry weight of extract (mg QE/g).

### 2.6. Free Radical Scavenging Activity

The radical scavenging activity was observed to demonstrate the antioxidant ability of plant extracts as described previously with slight modifications [32]. The reaction was done in 200 *μ*L volume by mixing 100 *μ*L DPPH (0.1 mM) and 100 *μ*L plant extract. Then, it was incubated for 25 minutes in the dark and absorbance was taken at 517 nm. The percent scavenging was calculated by the following formula:

(1) $$\begin{array}{c} \%\text{ scavenging}=\frac{A_{\text{o}}-A_{\text{t}}}{A_{\text{o}}}\times 100, \end{array}$$

where *A*_o_ is absorbance of DPPH radical with 50% DMSO and *A*_t_ is absorbance of DPPH radical with test or reference sample.

### 2.7. *In Vitro α*-Glucosidase Inhibitory Assay

The competitive inhibition-based assay with *α*-glucosidase was carried out using a method adopted by Fouotsa *et al*. [33] and Aryal *et al.* [34] with slight modifications. Briefly, 20 *μ*L of plant extract prepared on 30% DMSO and 80 *μ*L of the *α*-glucosidase enzyme (0.5 U/mL) prepared in 50 mM phosphate saline buffer (pH 6.8) were added, and initial absorbance was taken at 410 nm in a microplate reader. Then, the microtiter plate was preincubated at 37°C for 15 minutes. 100 *μ*L of 1.4 mM substrate (pNPG) was added and incubated at 37°C for 25 minutes, and absorbance was measured. Acarbose was used as the reference compound, and each assay was performed in triplicate for validation. The percentage inhibition of *α*-glucosidase by plant extract was calculated by using the following formula:

(2) $$\begin{array}{c} \%\text{ enzyme inhibition}=\left[\frac{A_{\text{c}}-A_{\text{t}}}{A_{\text{c}}}\right]\times 100, \end{array}$$

where *A*_c_ is the absorbance of enzyme-substrate reaction with 30% DMSO and *A*_t_ is the absorbance of enzyme-substrate with plant extract.

### 2.8. *In Vitro α*-Amylase Inhibition Assay

The competitive inhibition-based assay with *α*-amylase was carried out by following the method adopted by Khadayat *et al.* [32] with slight modifications. 20 *μ*L of plant extracts in 50% DMSO and 80 *μ*L of porcine pancreas *α*-amylase (1.5 U/mL) in phosphate buffer of pH 7.0 were loaded, and initial absorbance was taken at 405 nm. The microtiter plate was incubated at 37°C for 15 minutes. Then, 100 *μ*L of 1 mM CNPG3, the substrate was added to initiate the reaction with incubation for 25 minutes, and the change in absorbance was monitored at 405 nm [32]. Acarbose was used as a reference compound. The percentage inhibition of *α*-amylase enzyme was estimated using equation (2) mentioned earlier.

### 2.9. Antibacterial Assay

The antibacterial activity of *A. catechu* extract against Gram-positive (*Staphylococcus aureus* ATCC 25923) and Gram-negative (*Escherichia coli* ATCC 25922, *Klebsiella pneumonia* ATCC 13883, *Salmonella typhi* ATCC 14028, and *Shigella sonnei* ATCC 25931) was assayed through Agar well diffusion method in a Mueller Hinton Agar (MHA) plate [35]. The inoculum of microorganisms in Mueller Hinton broth (MHB) adjusted to 0.5 McFarland equivalents was spread on the surface of the MHA plate using a sterile cotton swab. Then, wells were punched aseptically into the agar surface by using a sterile cork borer of 6 mm diameter and filled with 50 *μ*L (50 mg/mL) of plant extract prepared in 50% DMSO. The plates were allowed to diffuse at room temperature for 2 hours. 50 *μ*L of neomycin (1 mg/mL) and DMSO was used as positive and negative control, respectively. The zone of inhibition (ZoI) was determined by measuring the diameters of bacterial growth on plates after incubation at 37°C for 24 hours.

The extracts with maximum ZoI were further evaluated for minimum inhibitory concentration (MIC) and minimum bactericidal concentration (MBC) via the resazurin-based turbidimetric broth microdilution method [36]. The minimum concentration before the color change (from blue to pink) was taken as MIC value, and higher concentrations were streaked onto the MHA plates at different compartments and incubated overnight at 37°C to observe concentration corresponding to no growth of bacteria, i.e., MBC.

### 2.10. LC-HRMS Analysis

The LC-HRMS analysis of ethyl acetate and the aqueous fraction was performed using an Agilent 6520, Accurate-Mass Q-TOF Mass Spectrometer equipped with a G1311A quaternary pump, G1329A autosampler, and G1315D diode array detector at Sophisticated Analytical Instrument Facility (SAIF), CSIR-Central Drug Research Institute, Lucknow. Source and scan parameter settings include gas temp: 30°C, gas flow: 11.01/min, nebulizer: 40 psi, VCap: 3500, fragmentor: 175, skimmer1: 65.0, and octopoleRF Peak: 750. The solvent elution consists of acetonitrile, 5 mM acetate buffer, and water at the flow rate of 1.5 mL/min. The elution gradient was started from 5% acetonitrile for 0.1 min to 30% acetonitrile for 10 min, 80% acetonitrile for 32 minutes, and back to its initial conditions. During the whole process, column temperature was maintained at 30°C. After passing through the flow cell of the diode array detector, the column elute was directed to Q-TOF HRMS fitted with an electrospray interface. The mass spectrum analysis was carried out using positive electron spray ionization (ESI-positive mode) within the mass range of 100-2000 daltons at a scan rate of 1.03.

### 2.11. Data Analysis

The results were processed by using Gen5 Microplate Data Collection and Analysis Software and then by MS Excel. Inhibition of enzymatic hydrolysis of the substrates (pNPG and CNPG3) by 50% (IC_50_) was calculated using the GraphPad Prism Software version 8. All the experiments were carried out in triplicate, and data were presented in mean ± standard deviation. Raw data files acquired from the LC-HRMS were processed using MZmine 2 and then Mestre Nova 12.0 for compound annotation using PubChem, Dictionary of Natural Products 2, ChemSpider, and METLIN database.

## 3. Results

### 3.1. Total Phenolic and Flavonoid Content

The TPC and TFC were expressed as the GAE/g and QE/g of extract using calibration curves of gallic acid and quercetin, respectively. The TPC of *A. catechu* was found to be 175.48 ± 4.67 mg GAE/g which was greater than its TFC examined, i.e., 7.66 ± 1.0 mg QE/g.

### 3.2. Free Radical Scavenging Activity

The antioxidant of ethanolic extracts of *A. catechu* was evaluated by using a quick, reliable, and reproducible method through the measurement of 2,2-diphenyl-1-picrylhydrazyl (DPPH) radical scavenging. DPPH radical scavenging results were reported as IC_50_ and compared with the IC_50_ value of quercetin (6.3 ± 1.0 *μ*g/mL) as a standard. The radical scavenging ability of plant extract is 23.76 ± 1.57 *μ*g/mL.

### 3.3. *α*-Glucosidase and *α*-Amylase Inhibitory Activities

The results of *α*-glucosidase and *α*-amylase inhibitory activity of the extracts are expressed in Table 1. Among the tested fraction, aqueous and ethyl acetate fraction showed the most activity with an IC_50_ value of 82.6 ± 0.3 and 130.2 ± 0.6 *μ*g/mL against the *α*-glucosidase as compared to acarbose (IC_50_ = 344.23 ± 1.03 *μ*g/mL). In the *α*-amylase assays, the crude ethanol extract showed antidiabetic activity with an IC_50_ of 67.8 ± 1.8 *μ*g/mL, compared to acarbose (IC_50_ = 6.1 ± 0.1 *μ*g/mL).

### 3.4. Antibacterial Activity

The antibacterial activity of plant extracts against *S. aureus* ATCC 25923, *E. coli* ATCC 25922, *K. pneumoniae* ATCC 13883, *S. typhi* ATCC 14028, and *S. sonnei* ATCC 25931 was performed. The antibacterial activity was measured in terms of ZoI diameter in millimeters (mm) as shown in Table 2. Based on the ZoI, MIC and MBC were evaluated against *S. aureus* ATCC 25923. The MIC and MBC of the aqueous fraction of *A. catechu* bark extract were 6.25 and 12.5 mg/mL while those of neomycin are 0.0625 and 0.25 mg/mL respectively.

### 3.5. LC-HRMS-Driven Molecular Annotation

**Figure 1:**
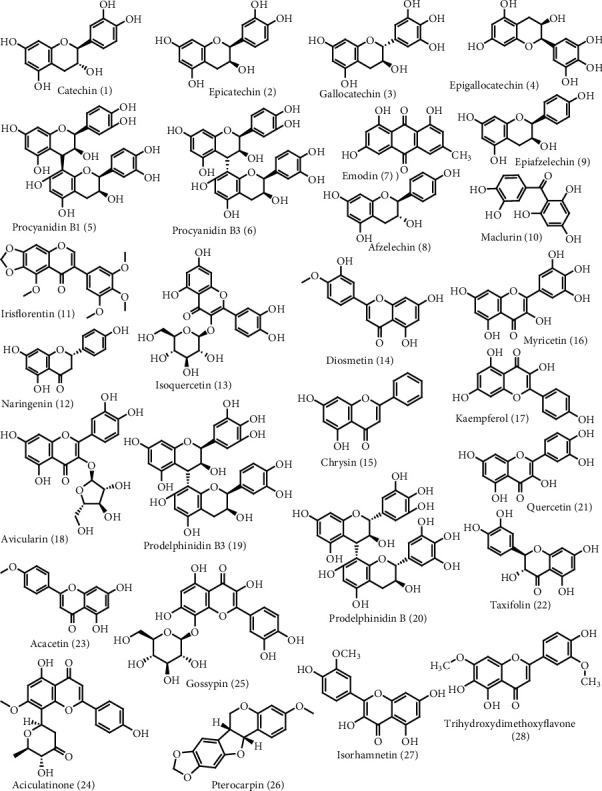
Annotated secondary metabolites in the ethanolic *A. catechu* bark extracts

The raw data of LC-HRMS were processed using MZmine, and the fraction with the best total ion chromatogram (TIC) was proceeding for further analysis using MestreNova 12.0 software. The TIC obtained using MZmine of ethyl acetate and aqueous fraction is shown in Figure S1. Details of identified compounds with their theoretical and observed mass to charge ratio, double bond equivalence (DBE), molecular formula, absolute errors in parts per million (ppm), and retention time (Rt) in positive ion mode in ESI are presented in Table 3. The compounds were identified based on the observed mass spectra and also compared with the literature data (Figure 1). We observed the presence of flavonoids and phenolic compounds in the extract of *A. catechu* such as catechin/epicatechin (*m*/*z* = 291.08) (Figure S2), gallocatechin/epigallocatechin (*m*/*z* = 307.08) (Figure S3), procyanidin B1/procyanidin B3 (*m*/*z* = 579.15) (Figure S4), emodin (*m*/*z* = 271.06) (Figure S5), epiafzelechin/afzelechin (*m*/*z* = 275.08) (Figure S6), maclurin (*m*/*z* = 263.05) (Figure S7), irisflorentin (*m*/*z* = 387.10) (Figure S8), naringenin (*m*/*z* = 273.07) (Figure S9), isoquercetin (*m*/*z* = 465.10) (Figure S10), diosmetin (*m*/*z* = 301.07) (Figure S11), chrysin (*m*/*z* = 255.06) (Figure S12), myricetin (*m*/*z* = 319.04) (Figure S13), kaempferol (*m*/*z* = 287.05) (Figure S14), avicularin (*m*/*z* = 435.09) (Figure S15), prodelphinidin B3 (*m*/*z* = 595.14) (Figure S16), prodelphinidin B (*m*/*z* = 611.14) (Figure S17), quercetin (*m*/*z* = 303.05) (Figure S18), taxifolin (*m*/*z* = 305.06) (Figure S19), acacetin (*m*/*z* = 285.07) (Figure S20), aciculatinone (*m*/*z* = 413.12) (Figure S21), gossypin (*m*/*z* = 481.09) (Figure S22), pterocarpin (*m*/*z* = 299.09) (Figure S23), isorhamnetin (*m*/*z* = 317.06) (Figure S24), and trihydroxy dimethoxyflavone (*m*/*z* = 331.08) (Figure S25).

## 4. Discussion

**Figure 2:**
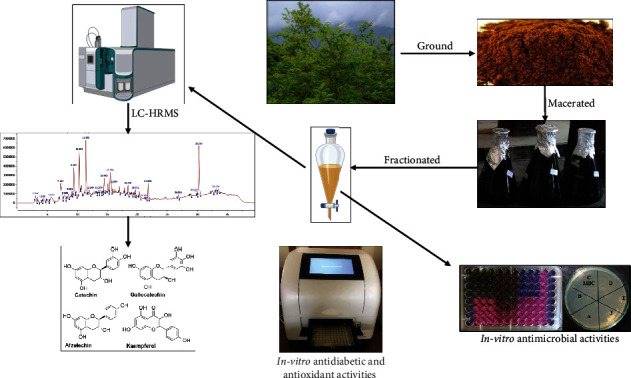
Schematic workflow of the study

The study was aimed at making a comparative study of ethanolic and methanolic extract of *A. catechu* in terms of antidiabetic, antioxidant, antibacterial, and secondary metabolite profiling.  Figure 2 shows the schematic workflow of the study. The TPC, TFC, and antioxidant activity of the crude ethanol extract of *A. catechu* were determined and compared with our previous work [34]. Studies have shown the valuable uses of plant-based bioactive secondary metabolites to mankind [8]. Plant extract contains many phenolic compounds with inhibitory activity against *α*-glucosidase supported by TPC and TFC of crude extract 175.48 ± 4.67 mg GAE/g and TFC 7.66 ± 1.0 mg QE/g respectively. The methanolic extract of *A. catechu* exhibited TPC and TFC of 186.675 ± 2.021 mg GAE/g and 10.24 ± 0.69 mg QE/g respectively [34]. Previous studies suggest that variation in TPC and TFC is due to differences in extracting solvents [70]. Likewise, the difference between antioxidant activity among the extracts might be due to the presence of metabolites at a different level which stabilizes the free radicals by donating hydrogen atoms [71, 72].

Compounds such as caprylic acid, methyl ester, lauric acid methyl ester, 2-ethyl-3-methyl-1-butene, myristic acid methyl ester [73], catechin, acacatechin, catechutannic acid, 4-hydroxybenzoic acid, afzelechin, epiafzelechin, mesquitol, ophioglonin, aromadendrin, kaempferol, baicalin, baicalein, and quercetin [74–77] and 5-hydroxy-2-[2-(4-hydroxyphenyl)acetyl]-3-methoxylbenzoic acid, (2S,3S)-3,7,8,3′,4′-pentahydroxyflavane, rhamnetin, 4-hydroxyphenyl ethanol, 3,3′,5,5′,7-pentahydroxyflavane, and fisetinidol [78] were isolated from *A. catechu*. Phytochemicals such as phenolic and flavonoid isolated from various parts of the plants are used as antioxidant, antimicrobial, antiulcer, antidiabetic, anticancer, antihyperlipidemic, antidiabetic, and hepatoprotective candidates [79, 80]. Previously, the IC_50_ value for inhibition of *α*-amylase activity by *A. catechu* ethanol extract was reported as 341.20 ± 15.30 *μ*g/mL by Lakshmi *et al.* [2], while ethanol extract showed an inhibition of 67.8 ± 1.8 *μ*g/mL in this study. Likewise, the methanolic extract of the *A. catechu* bark showed an IC_50_ of 115 ± 4.0 mg/mL and 23.7 ± 0.7 mg/mL against *α*-glucosidase and *α*-amylase, respectively [34], whereas leaf extracts showed an IC_50_ of 0.4977 mg/mL against *α*-glucosidase [81]. The difference in activities might be due to expression levels of secondary metabolites, which are greatly influenced by the regulation of biosynthetic gene clusters in climatic variation. Here, in our study, the crude extracts showed a potent inhibitory activity against carbohydrate hydrolases than that of the solvent fractions, which might be due to the synergistic effect of metabolites [82].

The phytochemicals exhibit a wide range of antioxidant, antidiabetic, and antimicrobial activity [83, 84]. The antimicrobial activity of extracts from different parts of *A. catechu* [85–87] has been shown previously. *A. catechu* resin exhibits an inhibitory effect against *B. subtilis* (MIC: 20 *μ*g/mL), *S. aureus* (MIC: 40 *μ*g/mL), *P. aeruginosa* (MIC: 220 *μ*g/mL), and *E. coli* (MIC: 330 *μ*g/mL) [87]. In our study, the fractions of *A. catechu* from ethanol extract showed comparable antibacterial activities to methanol extract fraction as reported by Aryal *et al.* [34]. It might be due to the same experimental conditions, the only difference in the extracting solvent.

The antioxidant mechanisms of flavonoid and phenolic compounds play an important role in protecting humans against infections and degenerative diseases [88]. Polyphenols with the number of hydroxyl groups can act as a source of hydrogen and electron donor to radicals to stabilize them and hence reduce the oxidative stress which plays an important role in the management of diabetes [89, 90]. Hydroxyl groups at 3′, 4′, and 5′of ring B (a pyrogallol group) and the double bond between carbon-2 and carbon-3 conjugated with the 4-oxo (=O) and 3-hydroxyl (-OH) group in ring C enhance the radical scavenging activity of flavonoids [91]. From the study of a structure-activity relationship, hydroxyl groups and their configuration, a ketonic functional group at carbon-4, and a double bond at carbons (C2–C3) are quite important structural features on flavonoids to show their antioxidant and antidiabetic ability [92, 93].

Isolation of plant metabolites is considerably challenging due to the lack of standardized instruments, income sources, and the availability of the laboratory in our context. To identify the metabolites within a short instance of time with a small number of samples, LC-HRMS of ethyl acetate and aqueous fractions of *A. catechu* bark extract were carried out. To further evaluate the metabolites, MZmine 2 was used to study the peak features of the raw MS files. The TIC obtained by overlaying through MZmine 2 is shown in Figure S1. Then, Mesternova 12.0 software is utilized to annotate the metabolites based on *m*/*z*, retention time, and molecular formula, and other databases are used to search and assign formulas and compound structures. Twenty-eight phenolic and flavonoids were annotated during our study by comparing our spectral data with the literature. The base peak at*m*/*z* 291.08, molecular formula C_15_H_14_O_6_, and DBE 9 and fragment peaks at 313.07 [M + Na]^+^ and 139.03 are speculated as catechin (**1**) or epicatechin (**2**). This data is consistent with Shen *et al*. [8] and Ibrahim *et al.* [37]. The fragmentation pattern of catechin and epicatechin is shown in Figure S26. Catechin/epicatechin was already reported with antidiabetic [94] (IC_50_ of 160 ± 67 *μ*g/mL against *α*-amylase; 31 *μ*g/mL and >290 *μ*g/mL against *α*-glucosidase), antimicrobial [95], anti-inflammatory [96], and antioxidant [97] abilities and also reduces the risk of ischemic heart disease [98]. Likewise, [M + H]^+^ at *m*/*z* 307.08, molecular formula C_15_H_14_O_7_, DBE 9, and along with fragment peak at 289.07 and 139.03 is considered as gallocatechin (**3**) or epigallocatechin (**4**) based on result analysis from Shen *et al.* [8]. The fragmentation pattern of gallocatechin/epigallocatechin is shown in Figure S27. It has shown different pharmacological activities such as antiviral [99, 100], antioxidant [101], and antibacterial [102] activities. Compounds with characteristic fragment ions 427.10 [M + H − 152]^+^ and 289.07 (kaempferol) and base peak [M + H]^+^ at *m*/*z* 579.15, molecular formula C_30_H_26_O_12_, and DBE 18 are annotated as procyanidin B1 (**5**) or procyanidin B (**6**). The spectral data coincides with data from Shen *et al.* [8]. Likewise, it is reported to be used as antioxidant, antibacterial, antiviral, anticarcinogenic, anti-inflammatory, antiallergic, and vasodilatory candidate. They can inhibit lipid peroxidation, platelet aggregation, and capillary hyperpermeability [62, 103]. The base peak [M + H]^+^ at *m*/*z* 271.06, molecular formula C_30_H_26_O_12_, and DBE 11 with characteristic fragment ions 253.16, 243.17, 229.14, 225.13, and 197.08 is explicated as emodin (**7**). The data is in agreement with Zhan *et al.* [42]. The fragmentation pattern of emodin is shown in Figure S28. Emodin also exhibits a wide spectrum of pharmacological properties including antiallergic, antiosteoporotic, antidiabetic, anti-inflammatory, anticancer, antiviral, antimicrobial, antioxidant, hepatoprotective, and immunosuppressive activities [104]. The compound with base peak 275.08, molecular formula C_15_H_14_O_5_, and DBE 9 and fragment peaks at 257.17, 233.08, 191.07, and 169.11 is expected to be afzelechin (**8**) or epiafzelechin (**9**). The data is consistent with Mittal *et al.* [43]. It has been reported to have antidiabetic [105], antimicrobial [106], and antioxidant [107] abilities.

The base peak at *m*/*z* 263.05 with molecular formula C_13_H_10_O_6_ and DBE 9 and fragment peaks at 153.08 are assigned as maclurin (**10**), consistent with the result of Berardini *et al.* [46]. Maclurin (**10**) has been reported to have antioxidant [108] and anticancer activities [109]. Similarly, the compound at base peak *m*/*z* 387.1 and fragment peaks at 357.09 (M + H − CH_3_ × 2)^+^, 372 [M + H − CH_3_]^+^ is assigned as irisflorentin (**11**) based on result analysis by Zhang *et al.* [49]. It is used as anti-inflammatory [110], antiallergic [111], anticholinesterase [112], and antimicrobial agent [113]. Likewise, mass spectrum with a base peak at *m*/*z* 273.07, molecular formula C_15_H_12_O, and DBE 10 and fragment peak at 153.10 is predicted to be naringenin (**12**) corresponding to fragment pattern analyzed by Tong *et al.* [50]. The fragmentation pattern of naringenin is shown in Figure S29. It shows antibacterial [114], anticancer [115], antioxidant, anti-inflammatory, hepatoprotective, nephroprotective, immunomodulatory, and antidiabetic properties [116]. Similarly, a base peak with *m*/*z* 465.1 and molecular formula C_21_H_20_O_12_ and the fragment ion at 303.05 and 289.07 are interpreted as isoquercetin (**13**) comparing our spectra with Liu *et al.* [51]. The fragmentation pattern of isoquercetin is shown in Figure S30. It acts as antioxidation, anticancer, anticardiovascular, antidiabetes, and antiallergic candidate [117]. Likewise, the compound with fragment peaks at 289.09 and 149.09 is annotated as diosmetin (**14**) having a base peak at *m*/*z* 301.07. The spectral data matches with data from Chen *et al*. [52] and Park *et al.* [53]. The fragmentation pattern of diosmetin is shown in Figure S31. It is used as an anticancer, anti-inflammatory, antioxidant, and antimicrobial agent [118]. The base peak at *m*/*z* 255.06, molecular formula C_15_H_10_O_4_, and DBE 11 and characteristic fragment ion at 153.12 are expected to be chrysin (**15**) with reference from the data collected from our spectra and Zhao *et al.* [54]. The fragmentation pattern of diosmetin is shown in Figure S32. It shows anticancer, antidiabetic, neuroprotective, antiallergic, antihyperlipidemic, antimicrobial, antiobesity, anti-inflammatory, hepatoprotective, cardiovascular, reproductive, and antioxidant activities [119]. The other flavonoid is expected to be myricetin (**16**) with base peak *m/z* 319.04, molecular formula C_15_H_10_O_8,_ and DBE 11 with reference from the data collected from our spectra and Saldanha *et al.* [55]. It is used as antimicrobial, antioxidant, and anti-inflammatory agent [120, 121]. Likewise, a base peak with *m*/*z* 287.05, molecular formula C_15_H_10_O_6_, and DBE 11 and fragment peak at *m*/*z* 259.13, 165.09, and 153.12 were predicted to be kaempferol (**17**) as spectral figures match with the literature of March and Miao [56]. The fragmentation pattern of kaempferol is shown in Figure S33. It has neuroprotective, antimicrobial, antioxidant, anti-inflammatory, and anticancer effects [122]. Moreover, in our spectra, a base peak with *m*/*z* 435.09, molecular formula C_21_H_20_O_13_, and DBE 12 and fragment peak at *m*/*z* 303.05, 287.06, and 183.10 were annotated as avicularin (**18**) taking reference of Santos *et al.* [57]. The fragmentation pattern of avicularin is shown in Figure S34. It is used as an anticancer, anti-inflammatory, and anti-infectious candidate [123].

The molecular ion peak at *m*/*z* 595.14, molecular formula C_30_H_26_O_13_, and DBE 18 and fragment ions at *m*/*z* 427.08, 169.07, 291.09, and 305.06 were annotated as prodelphinidin B3 (**19**). These spectral data are consistent with Friedrich *et al.* [40] and Pinto *et al.* [58]. The fragmentation pattern of prodelphinidin B3 is shown in Figure S35. It is used as antidiabetic, antiviral, and anti-inflammatory activities [124]. Additionally, [M + H]^+^ at *m*/*z* 611.14, molecular formula C_30_H_26_O_14_, and DBE 18 and fragment ions at *m*/*z* 307.08 are considered as prodelphinidin B (**20**) relying on result analysis from Navarro *et al.* [59]. The fragmentation pattern of prodelphinidin B is shown in Figure S36. It is used as antidiabetic, antiviral, and anti-inflammatory candidate [124]. Base peak *m*/*z* 303.05, molecular formula C_15_H_10_O_7_, DBE 11, and fragments ions at *m*/*z* 285.15, 257.13, 247.15, 229.13, 201.12, 183.10, 165.08, 153.11, 137.08, 121.03, and 111.06 were annotated as quercetin (**21**) whose spectra exactly match with the spectra data of Scigelova *et al.* [60]. It is used as an anti-inflammatory, antioxidant, antiviral, antimicrobial, and anticancer agent [125]. *A. catechu* may contain taxifolin (**22**) with the base peak at *m*/*z* 305.06, molecular formula C_15_H_12_O_7_, and DBE 10, and characteristic fragment ions are 287.05 (M + H − H_2_O)^+^ and 177.0253 (M + H − H_2_O − C_3_O_2_ − C_2_H_2_O]^+^ comparing our result with Michel *et al.* [61] and Yang *et al.* [62]. It is known to have potential antibacterial, antifungal, anti-inflammatory, analgesic, antioxidant, antipyretic, platelet inhibitory, and even anticancer actions [126, 127]. Likewise, a base peak at *m*/*z* 284.06, molecular formula C_16_H_12_O_5_, and DBE 11 and characteristic fragment ions at *m*/*z* 245.15 (M + H − CO_2_)^+^, 213.13(M − CO_2_ − CO + H)^+^ are annotated as acacetin (**23**). The data is the same as the spectral data of Yin *et al.* [63]. The molecular ion at *m*/*z* 413.12, molecular formula C_22_H_20_O_8_, and DBE 13 and fragments ions at *m*/*z* 325.10, 296.10, and 295.09 are characterized as aciculatinone (**24**). The spectral data exactly match with Shen *et al*.[64]. The base peak at *m*/*z* 481.09 and fragment peaks 319.04 which are in agreement with previous studies done by Giorio *et al.* [65] and Petsalo *et al*. [66] are speculated as gossypin (**25**). The base peak at *m*/*z* 299.09, molecular formula C_17_H_14_O_5_, and DBE 11 manifested that it could be pterocarpin (**26**) comparing spectral data with Geiger *et al*. [67]. Moreover, another annotated compound is isorhamnetin (**27)** with the base peak at *m*/*z* 317.06, molecular formula C_16_H_12_O_7_, and DBE 11. The data coincides with Chen *et al*. [68]. The fragmentation pattern of isorhamnetin is shown in Figure S37. Likewise, [M + H]^+^ at 331.08 along with fragment peak at 315.09, and 301.08 is considered as trihydroxy dimethoxyflavone (**28**) relying on result analysis from Zhang *et al*. [69]. The fragmentation pattern of trihydroxy dimethoxyflavone is shown in Figure S38.

In our study, we have annotated 28 secondary metabolites from the ethanol extract of *A. catechu* where seven of the secondary metabolites, namely, catechin, epicatechin, gallocatechin, epigallocatechin, procyanidin, emodin, and quercetin, were already annotated by Aryal *et al.* [34] in methanol extract. To support our annotation, more spectroscopic data are required. Hence, further investigation is required for the separation of potential enzyme inhibitors, antioxidant, and antibacterial compounds and hence the inhibitory activities and enzymatic kinetics of such compounds to develop them as future drug candidates or food supplements.

## 5. Conclusion

The bark of *A. catechu* has significant potential in inhibiting carbohydrate hydrolases due to the abundance of flavonoids and polyphenols. Our findings open the entryway for better utilization of phytoconstituents of *A. catechu* for the management of diabetes and pathogens. Further research on the isolation of potential inhibitors from solvent fraction, the study of pharmacokinetic parameters, kinetics, and *in vivo* experiments are required to develop an influential therapeutic approach against diabetes.

## Figures and Tables

**Table 1:** *α*-Glucosidase and *α*-amylase inhibitory activity of different fractions of *A. catechu*.

Extracts	IC_50_ (*μ*g/mL)	
	*α*-Glucosidase	*α*-Amylase
Crude ethanol extract	10.3 ± 0.1	67.8 ± 1.8
Hexane fraction	<50 %	<50 %
Dichloromethane fraction	174.7 ± 0.6	<50 %
Ethyl acetate fraction	130.2 ± 0.6	<50 %
Aqueous fraction	82.6 ± 0.3	<50%
Standard (acarbose)	344.2 ± 1.03	6.02 ± 0.1

**Table 2:** Zone of inhibition of each fraction of plants.

Microorganisms	Zone of inhibition (ZoI) in millimeters (mm)				
	Hexane Fraction	Dichloromethane fraction	Ethyl acetate fraction	Aqueous fraction	Positive control
*Staphylococcus aureus*	11 mm	9 mm	13 mm	14 mm	10 mm
*Escherichia coli*	—	—	—	—	16 mm
*Klebsiella pneumonia*	—	—	8 mm	10 mm	19 mm
*Salmonella typhi*	—	—	—	—	18 mm
*Shigella sonnei*	6 mm	7 mm	12 mm	10 mm	25 mm

Note: positive control (50 mg/mL neomycin).

**Table 3:** Identified compounds from *A. catechu* with names, base peak, double bond equivalence (DBE), retention time (Rt), and *m*/*z* values.

Annotated compounds	Systematic name	Calculated mass	Observed mass	Formula	DBE	Absolute error (ppm)	Rt (minute)	Fragment peak	Reference
Catechin (**1**)	[(2R,3S)-2-(3,4-Dihydroxyphenyl)-3,4-dihydro-2H-chromene-3,5,7-triol]	290.07	291.08	C_15_H_14_O_6_	9	0.16	10.4	313.07 [M + Na]^+^ and 139	[8, 37, 38] LC/MS and LC/MS/MS, NMR, UV
Epicatechin (**2**)	[(2*R*,3*R*)-2-(3,4-Dihydroxyphenyl)-3,4-dihydro-2*H*-chromene-3,5,7-triol]	290.07	291.08	C_15_H_14_O_6_	9	0.16	10.4	313.07 [M + Na]^+^ and 139.03	[8, 37, 38] LC/MS and LC/MS/MS, UV, NMR
Gallocatechin (**3**)	[(2*R*,3*S*)-2-(3,4,5-Trihydroxyphenyl)-3,4-dihydro-2*H*-chromene-3,5,7-triol]	306.07	307.08	C_15_H_14_O_7_	9	2.26	7.15	313.07 [M + Na]^+^, 289.07, and 139.03	[8, 38, 39] LC/MS and LC/MS/MS, UV, NMR
Epigallocatechin (**4**)	[(2*R*,3*R*)-2-(3,4,5-Trihydroxyphenyl)-3,4-dihydro-2*H*-chromene-3,5,7-triol]	306.07	307.08	C_15_H_14_O_7_	9	2.26	7.15	313.07 [M + Na]^+^, 289.07, and 139.03	[8, 38, 39] LC/MS and LC/MS/MS, UV, NMR
Procyanidin B1 (**5**)	(*2R,2*′*R,3R,3*′*S,4R*)-2,2′-Bis(3,4-dihydroxyphenyl)-3,3′,4,4′-tetrahydro-2H,2'H-4,8′-bichromene-3,3′,5,5′,7,7′-hexol	578.15	579.15	C_30_H_26_O_12_	18	2.21	9.87	427.10 [M + H − 152]^+^, 289.07 (kampferol)	[8, 40] HPLC, LC/MS, and LC/MS/MS
Procyanidin B3 (**6**)	(*2R,2*′*R,3S,3*′*S,4S*)-2,2′-Bis(3,4-dihydroxyphenyl)-3,3′,4,4′-tetrahydro-2H,2′H-4,8′-bichromene-3,3′,5,5′,7,7′-hexol	578.15	579.15	C_30_H_26_O_12_	18	2.21	9.87	427.10 [M + H − 152]^+^, 289.07 (kampferol)	[8, 41] NMR, (Q-Trap), LC/MS and LC/MS/MS
Emodin (**7**)	1,3,8-Trihydroxy-6-methyl-9,10-anthraquinone	270.05	271.06	C_15_H_10_O_5_	11	4.18	18.60	253.16, 243.17, 229.14, 225.13, and 197.08	[42] UPLC-DAD-MS/MS
Afzelechin (**8**)	(2*R*,3*S*)-2-(4-Hydroxyphenyl)-3,4-dihydro-2*H*-chromene-3,5,7-triol	274.08	275.08	C_15_H_14_O_5_	9	3.51	12.77	257.17, 233.08	[43–45] UHPLC-PDA-HRMS, HPLC/ESI-MS and NMR, HPLC/MS/MS
Epiafzelechin (**9**)	(2*R*,3*R*)-2-(4-Hydroxyphenyl)-3,4-dihydro-2*H*-chromene-3,5,7-triol	274.08	275.08	C_15_H_14_O_5_	9	3.51	12.77	257.17, 233.08	[43–45] UHPLC-PDA-HRMS, HPLC/ESI-MS and NMR, HPLC/MS/MS
Maclurin (**10**)	(3,4-Dihydroxyphenyl)-(2,4,6-trihydroxyphenyl)methanone	262.04	263.05	C_13_H_10_O_6_	9	4.24	2.96	153.08	[46] HPLC/ESI-MS
Irisflorentin (**11**)	9-Methoxy-7-(3,4,5-trimethoxyphenyl)-[1,3]dioxolo[4,5-g]chromen-8-one	386.09	387.1	C_20_H_18_O_8_	12	2.45	5.07	357.09 [M + H − CH_3_ × 2]^+^, 372.07 [M + H − CH_3_]^+^	[47–49] HPLC–DAD–ESI-MS, NMR, LC-MS
Naringenin (**12**)	5,7-Dihydroxy-2-(4-hydroxyphenyl)chroman-4-one	272.06	273.07	C_15_H_12_O_5_	10	2.07	12.56	153.03	[50] (LC–MS/MS)
Isoquercetin (**13**)	2-(3,4-Dihydroxyphenyl)-5,7-dihydroxy-3-[(2*S*,3*R*,4*S*,5*S*,6*R*)-3,4,5-trihydroxy-6-(hydroxymethyl)oxan-2-yl]oxychromen-4-one	464.09	465.1	C_21_H_20_O_12_	12	3.02	12.95	303.05 (quercetin), 289.07 (kampferol)	[51]
Diosmetin (**14**)	5,7-Dihydroxy-2-(3-hydroxy-4-methoxyphenyl)-4H-chromen-4-one	300.06	301.07	C_16_H_12_O_6_	11	4.94	13.72	289.09, 149.09	[52, 53] UHPLC-LTQOrbitrap MS, NMR
Chrysin (**15**)	5,7-Dihydroxy-2-phenylchromen-4-one	254.05	255.06	C_15_H_10_O_4_	11	2.26	16.18	153.12	[54] UPLC-MS/MS
Myricetin (**16**)	3,5,7-Trihydroxy-2-(3,4,5-trihydroxyphenyl)chromen-4-one	318.03	319.04	C_15_H_10_O_8_	11	3.54	12.64		[55] HPLC-PAD, UV/MS + NMR
Kaempferol (**17**)	3,5,7-Trihydroxy-2-(4-hydroxyphenyl)chromen-4-one	286.04	287.05	C_15_H_10_O_6_	11	1.16	17.17	259.13, 165.09, 153.12	[56] Q-TOF-HRMS
Avicularin (**18**)	3-[(2*S*,3*R*,4*R*,5*S*)-3,4-Dihydroxy-5-(hydroxymethyl)oxolan-2-yl]oxy-2-(3,4-dihydroxyphenyl)-5,7-dihydroxychromen-4-one	434.08	435.09	C_21_H_20_O_13_	12	3.39	13.38	303.05, 287.06, and 183.10	[57] UHPLC-DAD-ESI-HRMS/MS, NMR
Prodelphinidin B3 (**19**)	(*2R,2*′*R,3S,3*′*S,4S*)-2,2′-Bis(3,4,5-trihydroxyphenyl)-3,3′,4,4′-tetrahydro-2H,2′H-4,8′-bichromene-3,3′,5,5′,7,7′-hexol	594.13	595.14	C_30_H_26_O_13_	18	3.03	5.78	427.08, 169.07, 291.09, 305.07	[40, 58] HPLC, ESI-Q-TOF
Prodelphinidin B (**20**)	2-(3,4,5-Trihydroxyphenyl)-8-[3,5,7-trihydroxy-2-(3,4,5-trihydroxyphenyl)-3,4-dihydro-2*H*-chromen-4-yl]-3,4-dihydro-2*H*-chromene-3,5,7-triol	610.13	611.14	C_30_H_25_O_14_	18	2.52	8.05	307.08	[59] UPLC-ESI-MS
Quercetin (**21**)	2-(3,4-Dihydroxyphenyl)-3,5,7-trihydroxychromen-4-one	302.04	303.05	C_15_H_10_O_7_	11	1.6	13.96	285.15, 257.13, 247.15, 229.13, 201.12, 183.10, 165.08, 153.11, 137.08, 121.03, 111.06	[60] FTMS, HPLC, LC MS/MS, and LCMS
Taxifolin (**22**)	(2R,3R)-2-(3,4-Dihydroxyphenyl)-3,5,7-trihydroxy-2,3-dihydrochromen-4-one	304.05	305.06	C_15_H_12_O_7_	10	1.78	13.51	287.05, 179.09	[61, 62] 1UHPLC-DAD-FLD, UHPLC-HRMS/MS, and HPLC-ESI-IT-TOF-MS
Acacetin (**23**)	5,7-Dihydroxy-2-(4-methoxyphenyl)chromen-4-one	284.06	285.07	C_16_H_12_O_5_	11	2.64	16.38	245.15 (M + H − CO_2_)^+^, 213.13(M − CO_2_ − C0 + H)^+^	[63] UHPLC-Q-TOF-MS/MS
Aciculatinone (**24**)	5-Hydroxy-8-[(2*R*,5*R*,6*R*)-5-hydroxy-6-methyl-4-oxooxan-2-yl]-2-(4-hydroxyphenyl)-7-methoxychromen-4-one	412.11	413.12	C_22_H_20_O_8_	13	3.80	8.18	325.10,296.10, 295.09	[64] NMR, HREIMS
Gossypin (**25**)	2-(3,4-Dihydroxyphenyl)-3,5,7-trihydroxy-8-[(2*S*,3*R*,4*S*,5*S*,6*R*)-3,4,5-trihydroxy-6-(hydroxymethyl)oxan-2-yl]oxychromen-4-one	480.09	481.09	C_21_H_20_O_13_	12	2.32	11.79	319.04,	[65, 66] HRMS, LCMS/MS
Pterocarpin (**26**)	(1*R*,12*R*)-16-Methoxy-5,7,11,19-tetraoxapentacyclo[10.8.0.0^2,10^.0^4,8^.0^13,18^]icosa-2,4(8),9,13(18),14,16-hexaene	298.08	299.09	C_17_H_14_O_5_	11	3.59	14.59		[67] DART-TOF-MS
Isorhamnetin (**27)**	3,5,7-Trihydroxy-2-(4-hydroxy-3-methoxyphenyl)chromen-4-one	316.05	317.06	C_16_H_12_O_7_	11	1.03	17.70	303.21, 274.20, 153.12	[68] LC-MS/MS
Trihydroxy dimethoxyflavone (**28**)	5,6-Dihydroxy-2-(4-hydroxy-3-methoxyphenyl)-7-methoxychromen-4-one	330.07	331.08	C_17_H_14_0_7_	11	2.36	14.25	301.08 and 315.09	[69] UHPLC/Q-TOF MS

## Data Availability

The data used to support the findings of this study are available from the corresponding author upon request.

## Abbreviations

CNPG3: 2-Chloro-4-nitrophenyl-*α*-D-maltotrioside
DBE: Double bond equivalence
DM: Diabetes mellitus
DPPH: 2,2-Diphenyl-1-picryl hydrazyl
HRMS: High-resolution mass spectrometry
LC-HRMS: Liquid chromatography-high resolution mass spectrometry
MBC: Minimum bactericidal concentration
mg QE/g: Milligrams of quercetin equivalent per gram dry weight of extract
mg GAE/g: Milligrams of gallic acid equivalent per gram dry weight of extract
MHA: Mueller Hinton Agar
MIC: Minimum inhibitory concentration
MS: Mass spectrometry
pNPG: 4-Nitrophenyl-*α*-D-glucopyranoside
Rt: Retention time
TFC: Total flavonoid contents
TIC: Total ion chromatogram
TPC: Total phenolic contents
ZoI: Zone of inhibition.

## Supplementary Materials

Supplementary Materials Table S1: Indigenous uses and chemical constituents' description of *A. catechu* used in the study. Figure S1: Total ion chromatogram (TIC) blue line for *A. catechu* ethyl acetate fraction and red line for *A. catechu* water fraction. Figure S2: Mass spectrum of catechin or epicatechin. Figure S3: Mass spectrum of gallocatechin/epigallocatechin. Figure S4: Mass spectrum of procyanidin B1/procyanidin B3. Figure S5: Mass spectrum of emodin. Figure S6: Mass spectrum of afzelechin/epiafzelechin. Figure S7: Mass spectrum of maclurin. Figure S8: Mass spectrum of irisflorentin. Figure S9: Mass spectrum of naringenin. Figure S10: Mass spectrum of isoquercetin. Figure S11: Mass spectrum of diosmetin. Figure S12: Mass spectrum of chrysin. Figure S13: Mass spectrum of myricetin. Figure S14: Mass spectrum of kaempferol. Figure S15: Mass spectrum of avicularin. Figure S16: Mass spectrum of prodelphinidin B3. Figure S17: Mass spectrum of prodelphinidin B. Figure S18: Mass spectrum of quercetin. Figure S19: Mass spectrum of taxifolin. Figure S20: Mass spectrum of acacetin. Figure S21: Mass spectrum of aciculatinone. Figure S22: Mass spectrum of gossypin. Figure S23: Mass spectrum of pterocarpan. Figure S24: Mass spectrum of isorhamnetin. Figure S25: Mass spectrum of trihydroxy dimethoxyflavone. Figure S26: Fragmentation pattern of catechin/epicatechin. Figure S27: Fragmentation pattern of gallocatechin/epigallocatechin. Figure S28: Fragmentation pattern of emodin. Figure S29: Fragmentation pattern of naringenin. Figure S30: Fragmentation pattern of isoquercetin. Figure S31: Fragmentation pattern of diosmetin. Figure S32: Fragmentation pattern of chrysin. Figure S33: Fragmentation pattern of kaempferol. Figure S34: Fragmentation pattern of avicularin. Figure S35: Fragmentation pattern of prodelphinidin B3. Figure S36: fragmentation pattern of prodelphinidin B. Figure S37: Fragmentation pattern of isorhamnetin. Figure S38: Fragmentation pattern of trihydroxy dimethoxyflavone.
