# Supplementary material for: LC-HRMS Profiling and Antidiabetic, Antioxidant, and Antibacterial Activities of Acacia catechu (L.f.) Willd
Source: Biomed Res Int. 2021 Aug 13;2021:7588711. doi: 10.1155/2021/7588711 (PMC8380500; doi:10.1155/2021/7588711)
Supplement: Supplementary Materials — Table S1: Indigenous uses and chemical constituents' description of A. catechu used in the study. Figure S1: Total ion chromatogram (TIC) blue line for A. catechu ethyl acetate fraction and red line for A. catechu water fraction. Figure S2: Mass spectrum of catechin or epicatechin. Figure S3: Mass spectrum of gallocatechin/epigallocatechin. Figure S4: Mass spectrum of procyanidin B1/procyanidin B3. Figure S5: Mass spectrum of emodin. Figure S6: Mass spectrum of afzelechin/epiafzelechin. Figure S7: Mass spectrum of maclurin. Figure S8: Mass spectrum of irisflorentin. Figure S9: Mass spectrum of naringenin. Figure S10: Mass spectrum of isoquercetin. Figure S11: Mass spectrum of diosmetin. Figure S12: Mass spectrum of chrysin. Figure S13: Mass spectrum of myricetin. Figure S14: Mass spectrum of kaempferol. Figure S15: Mass spectrum of avicularin. Figure S16: Mass spectrum of prodelphinidin B3. Figure S17: Mass spectrum of prodelphinidin B. Figure S18: Mass spectrum of quercetin. Figure S19: Mass spectrum of taxifolin. Figure S20: Mass spectrum of acacetin. Figure S21: Mass spectrum of aciculatinone. Figure S22: Mass spectrum of gossypin. Figure S23: Mass spectrum of pterocarpan. Figure S24: Mass spectrum of isorhamnetin. Figure S25: Mass spectrum of trihydroxy dimethoxyflavone. Figure S26: Fragmentation pattern of catechin/epicatechin. Figure S27: Fragmentation pattern of gallocatechin/epigallocatechin. Figure S28: Fragmentation pattern of emodin. Figure S29: Fragmentation pattern of naringenin. Figure S30: Fragmentation pattern of isoquercetin. Figure S31: Fragmentation pattern of diosmetin. Figure S32: Fragmentation pattern of chrysin. Figure S33: Fragmentation pattern of kaempferol. Figure S34: Fragmentation pattern of avicularin. Figure S35: Fragmentation pattern of prodelphinidin B3. Figure S36: fragmentation pattern of prodelphinidin B. Figure S37: Fragmentation pattern of isorhamnetin. Figure S38: Fragmentation pattern of trihydroxy dimethoxyflavone. [file 7588711.f1.docx]

**LC-HRMS Profiling and Antidiabetic, Antioxidant, and Antibacterial Activities of *Acacia catechu* (L.f.) Willd**

Babita Aryal^1#^, Bikash Adhikari^1#^_,_ Niraj Aryal^2^, Bibek Raj Bhattarai^1^, Karan Khadayat, and Niranjan Parajuli^1*^

*^1^Biological Chemistry Lab, Central Department of Chemistry, Tribhuvan University, Kirtipur, Kathmandu, Nepal*

*^2^Pharmaceutical Institute, Department of Pharmaceutical Biology, University of Tübingen, Germany*

**Table S1:** Indigenous uses and chemical constituents’ description of *A. catechu* used in the study

| **Scientific Name** | **Family** | **Indigenous uses** | **Voucher specimen** | **Chemical constituents** |
| --- | --- | --- | --- | --- |
| *A. catechu* | Fabaceae | Different parts of the plant extracts were known to have strong antioxidant, antimicrobial, anti-inflammatory, antipyretic, antidiarrheal, anti-hyperglycemic, hepatoprotective and immunomodulatory activity. Its extracts also show antiviral activity against dengue virus, human immunodeficiency virus, Herpes simplex virus, hepatitis B, and C virus. It has high medicinal values which are used for varied purposes like dysentery, colitis, gastric problems, asthma, cough, renal problems, leprosy, sore throat, gingivitis, dental and oral infections. Gum exudates from Acacia species have been widely used as a demulcent, emulsifiers, adhesives, and stabilizers in the food, textile, cosmetic, and soft drink industries. | TUCH-201011 | Caprylic acid methyl ester, lauric acid methyl ester, 2-ethyl-3-methyl-1-butene, myristic acid methyl ester, catechin, acacatechin, catechutannic acid, 4-hydroxybenzoic acid, afzelechin, epiafzelechin, mesquitol, ophioglonin, aromadendrin, kaempferol, epicatechin, baicalin, baicalein, and quercetin; 5-hydroxy-2-[2-(4-hydroxyphenyl)acetyl]-3-methoxylbenzoic acid, (2S,3S)-3,7,8,3′,4′-pentahydroxyflavane, rhamnetin, 4-hydroxyphenyl ethanol, 3,3′,5,5′,7-pentahydroxyflavane, and fisetinidol |


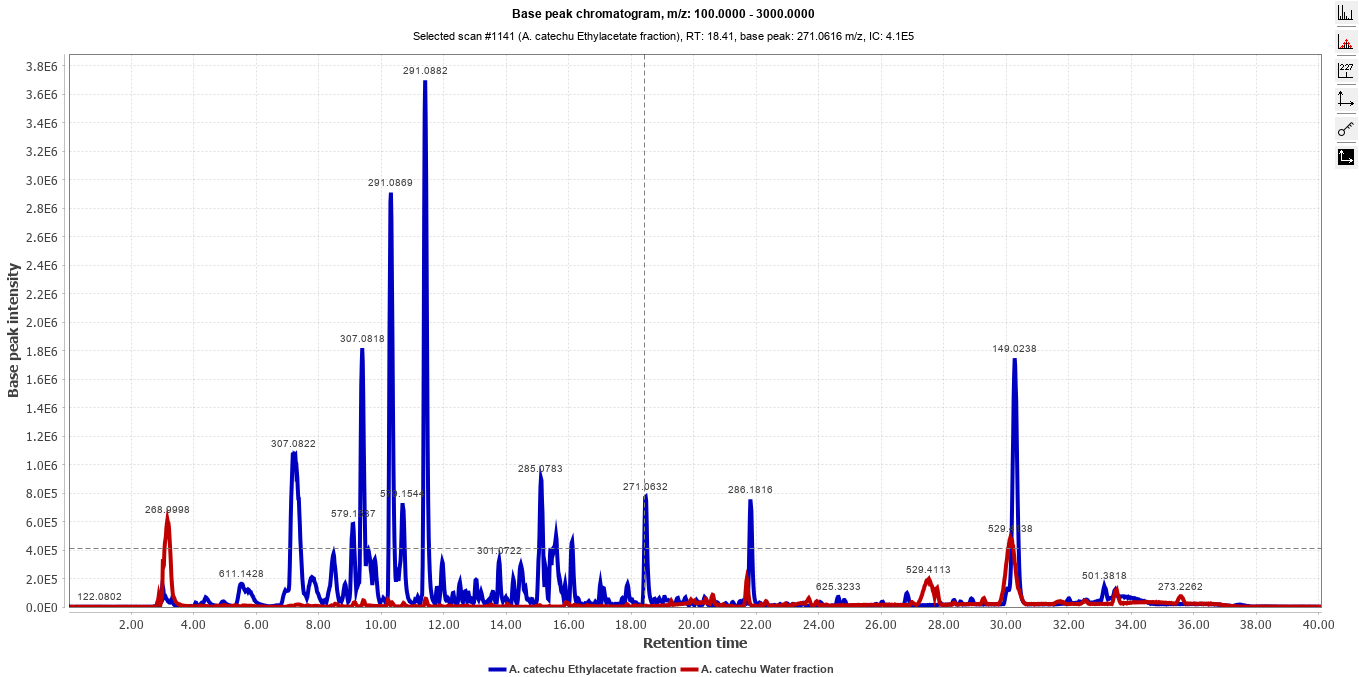


**Figure S1** Total ion chromatogram (TIC) blue line for *A. catechu* ethyl acetate fraction, and Red line for *A. catechu* water fraction


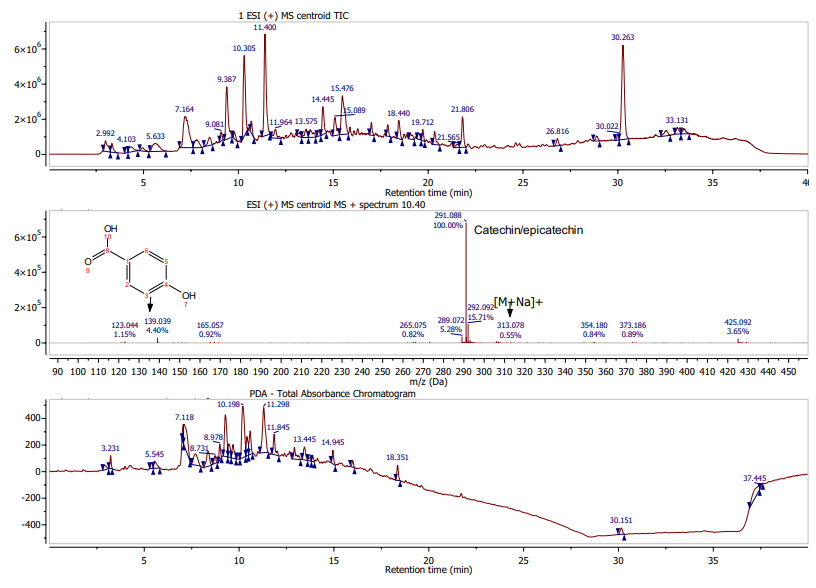


**Figure S2** Mass spectrum of catechin or epicatechin


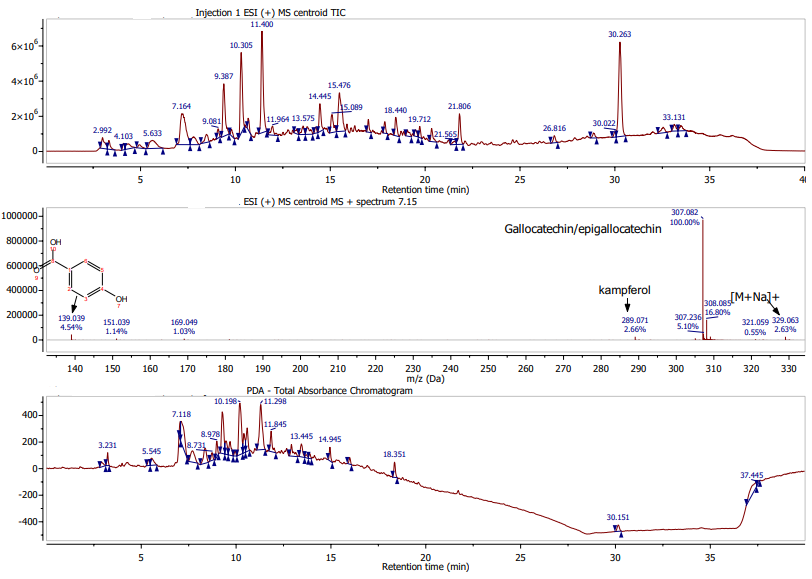


**Figure S3** Mass spectrum of gallocatechin/epigallocatechin


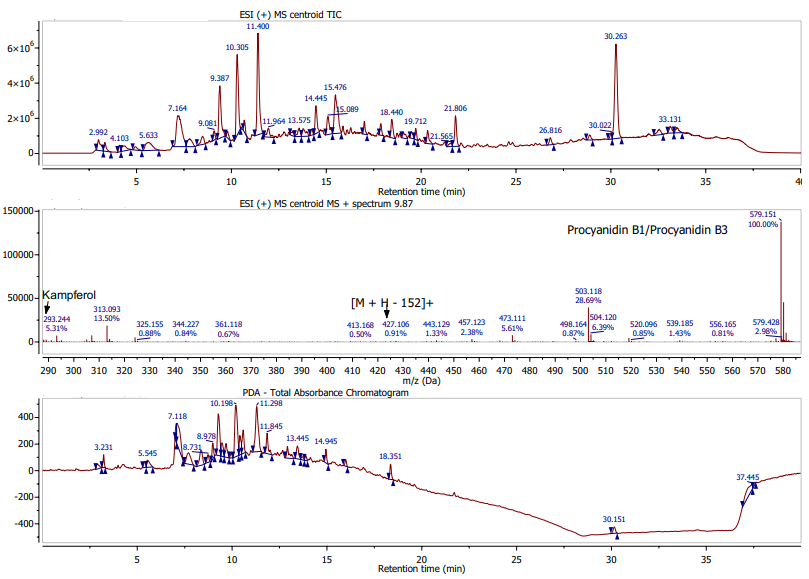


**Figure S4** Mass spectrum of procyanidin B1/procyanidin B3


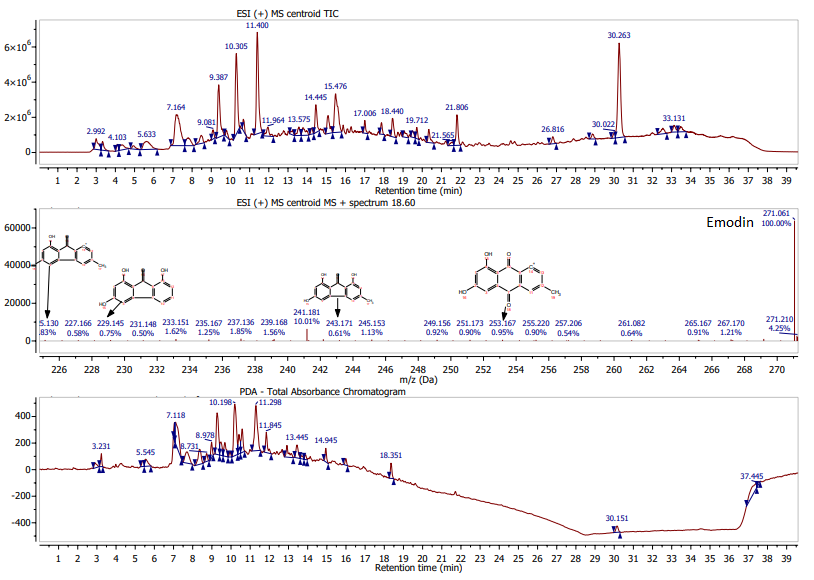


**Figure S5** Mass spectrum of emodin


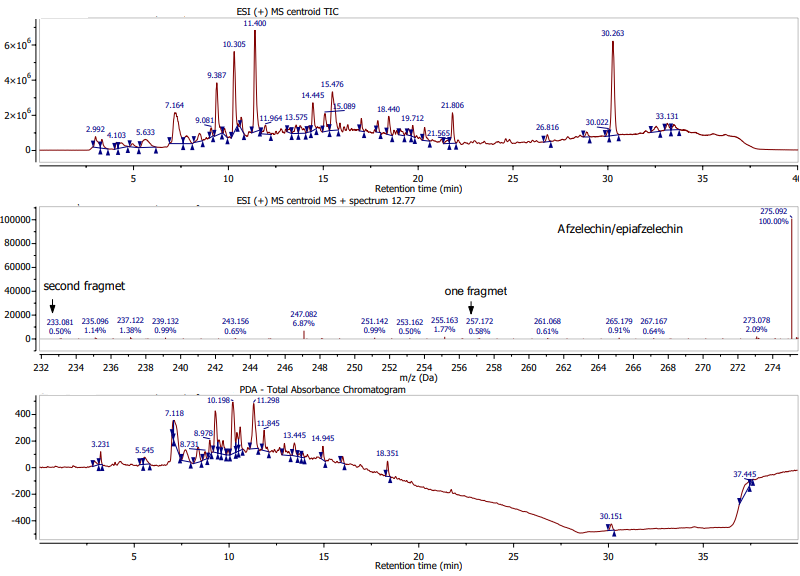


**Figure S6** Mass spectrum of afzelechin/epiafzelechin


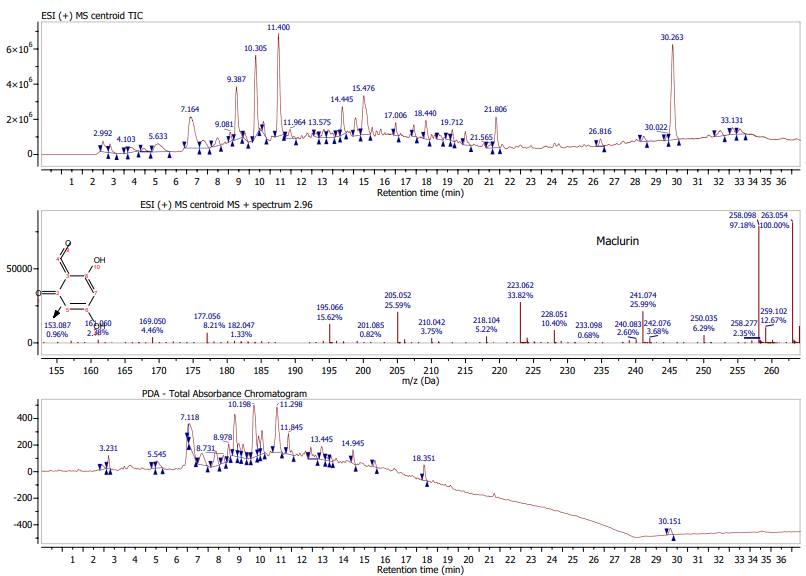


**Figure S7** Mass spectrum of [Maclurin](https://pubchem.ncbi.nlm.nih.gov/compound/Maclurin)


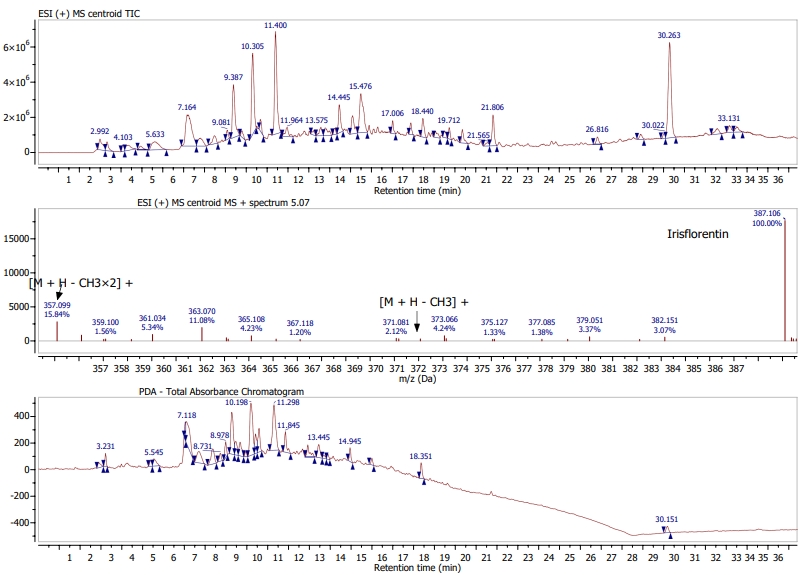


**Figure S8** Mass spectrum of irisflorentin


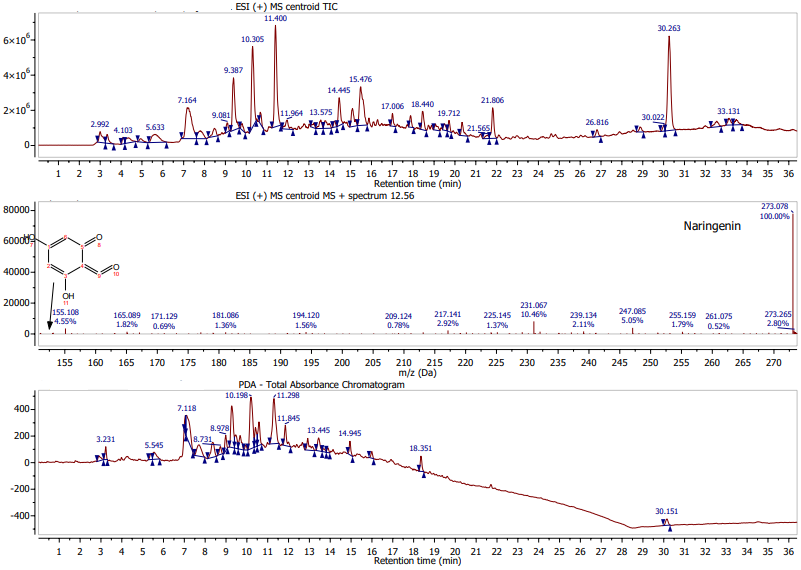


**Figure S9** Mass spectrum of naringenin


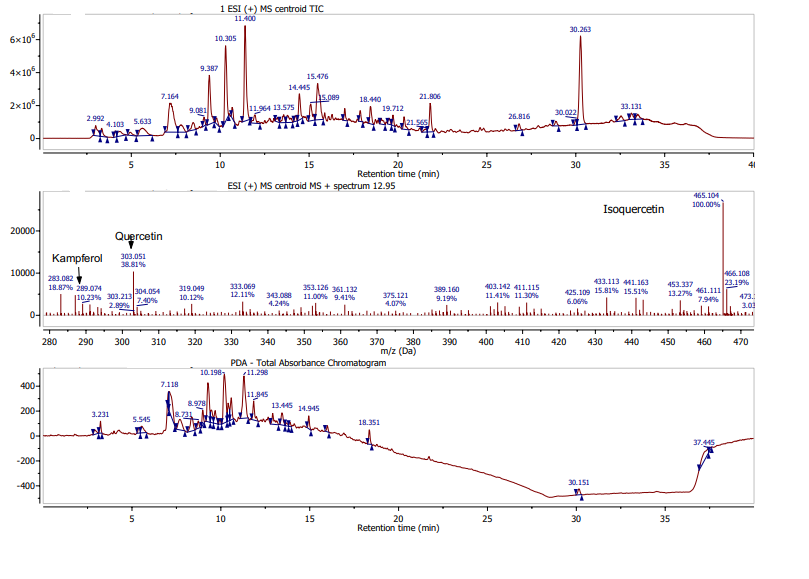


**Figure S10** Mass spectrum of isoquercetin


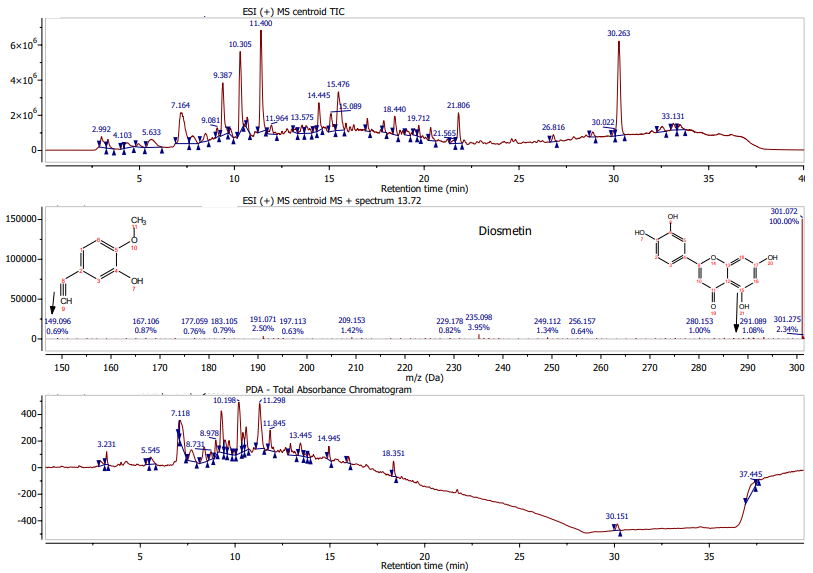


**Figure S11** Mass spectrum of diosmetin


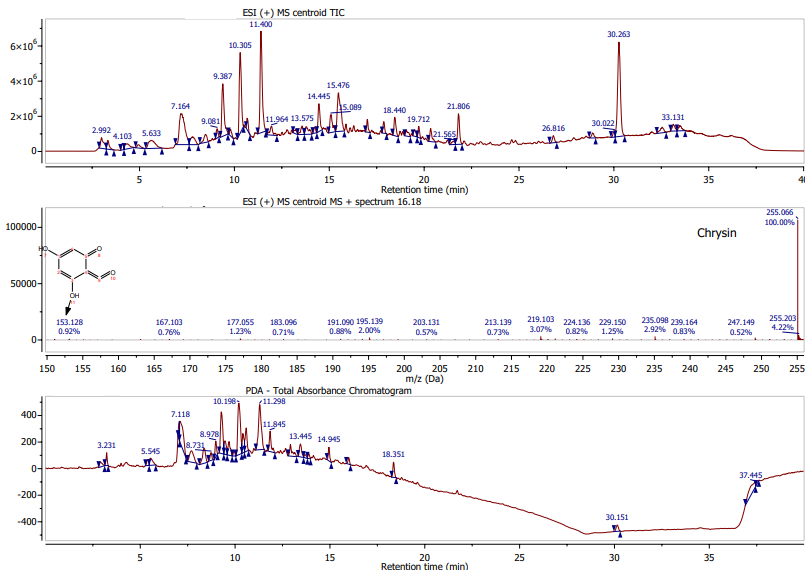


**Figure S12** Mass spectrum of chrysin


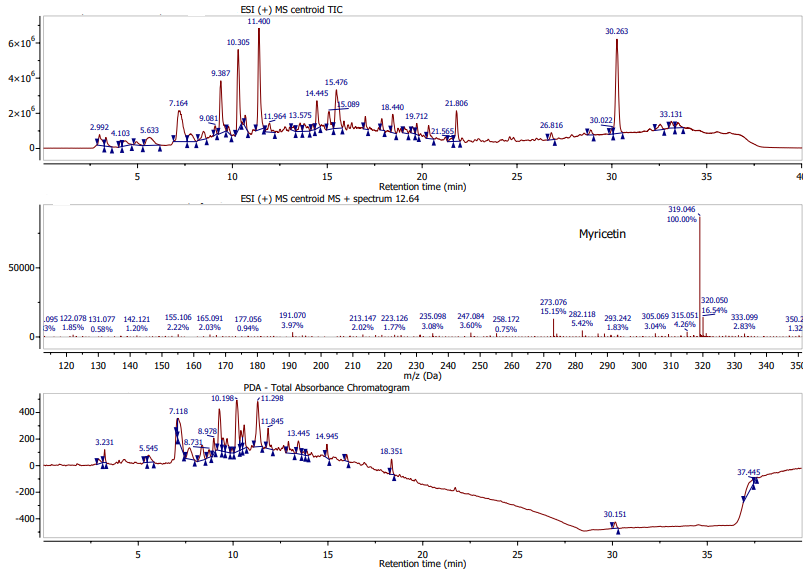
 **Figure S13** Mass spectrum of myricetin


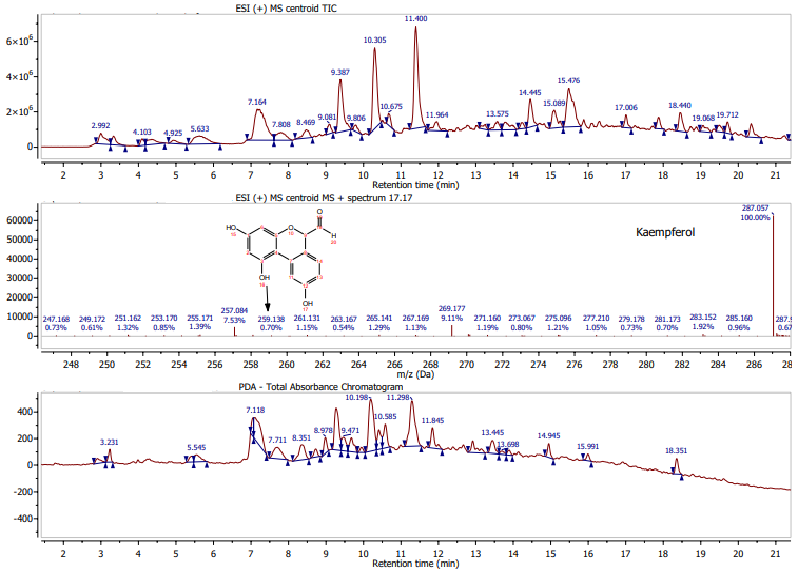


**Figure S14** Mass spectrum of kaempferol


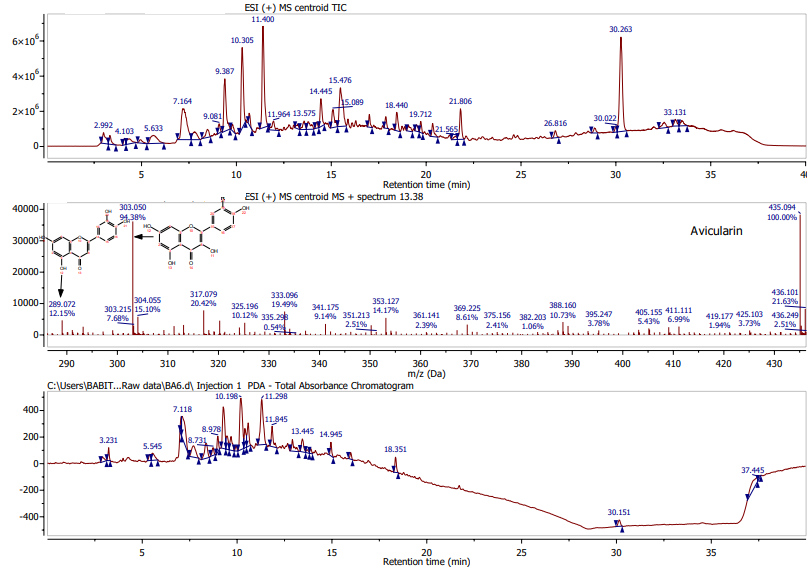
 **Figure S15** Mass spectrum of avicularin


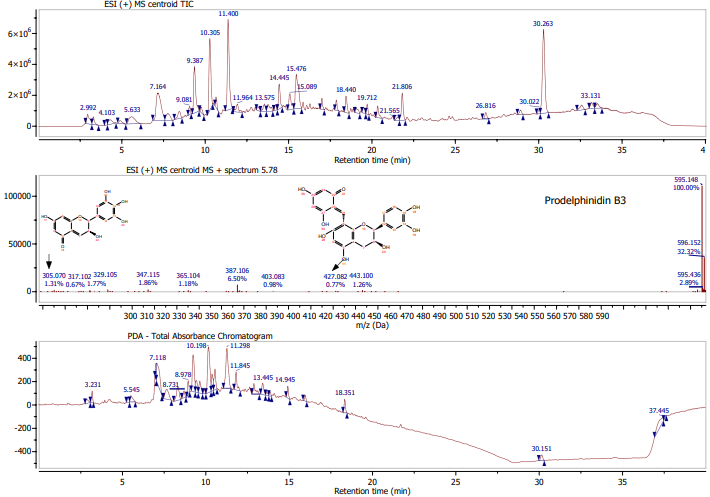
 **Figure S16** Mass spectrum of prodelphinidin B3


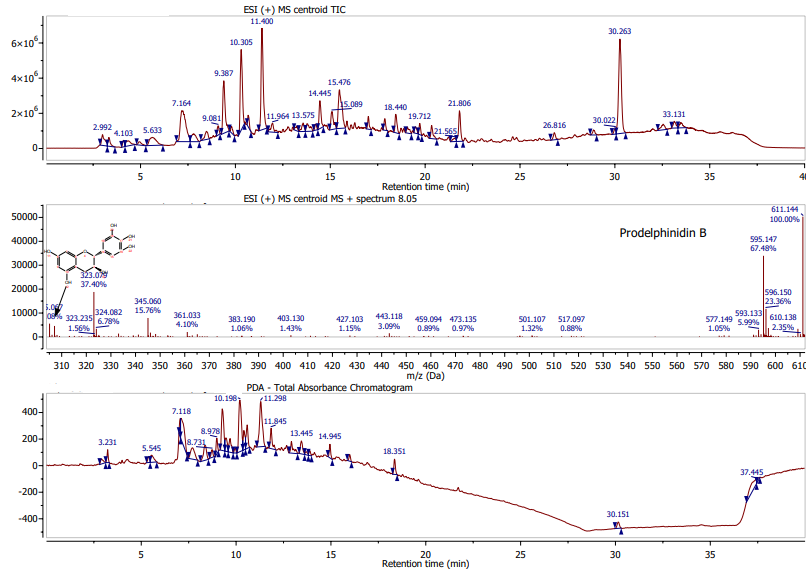


**Figure S17** Mass spectrum of prodelphinidin B


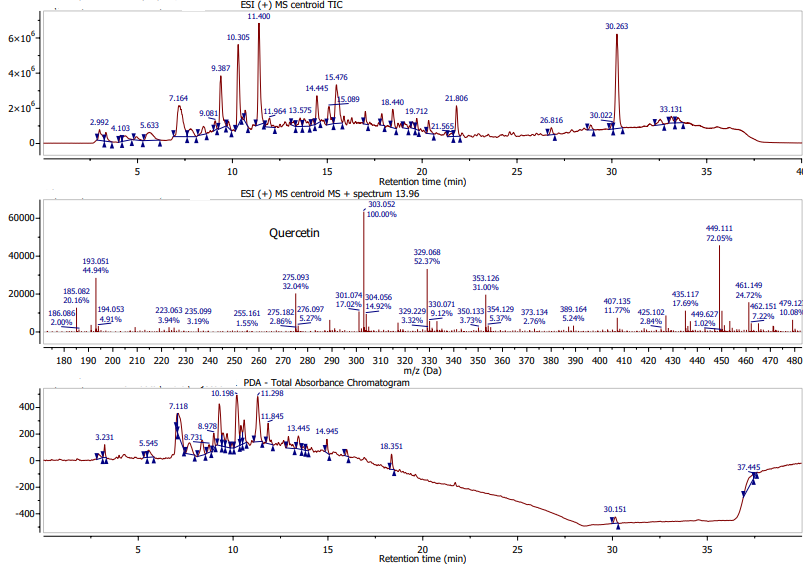


**Figure S18** Mass spectrum of quercetin


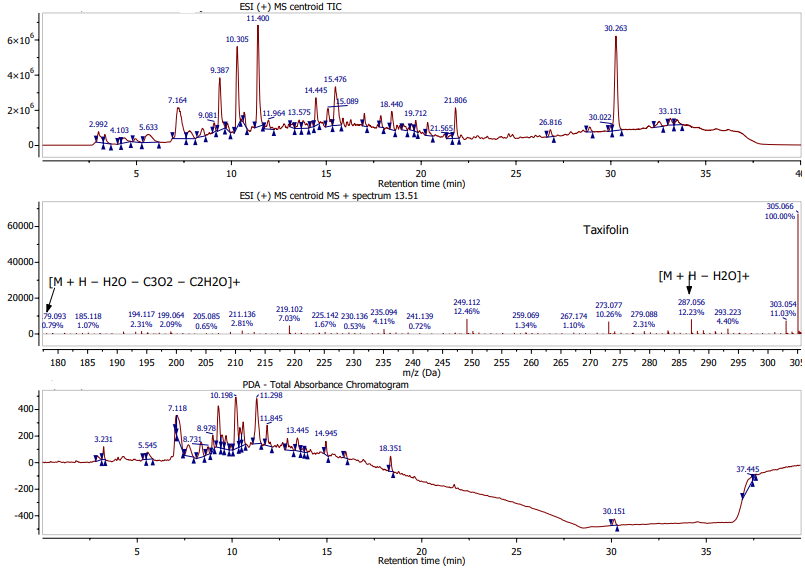


**Figure S19** Mass spectrum of [taxifolin](https://pubchem.ncbi.nlm.nih.gov/compound/Taxifolin)


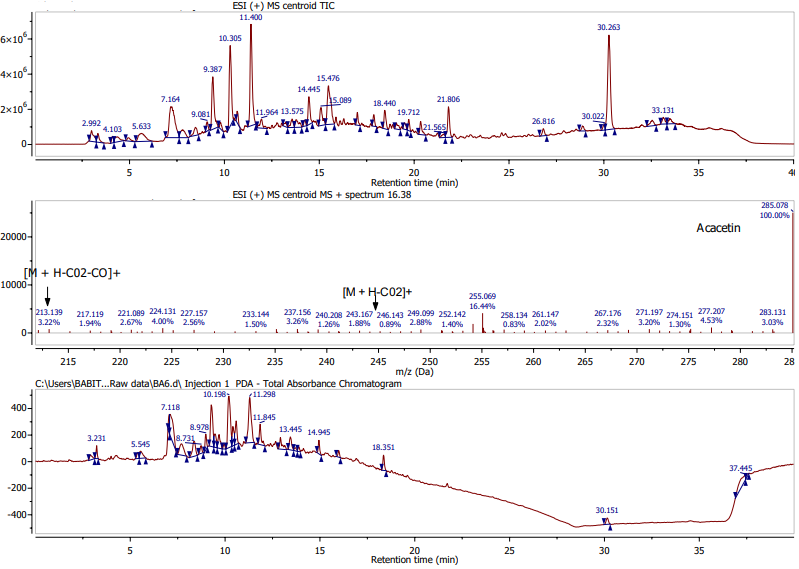


**Figure S20** Mass spectrum of acacetin


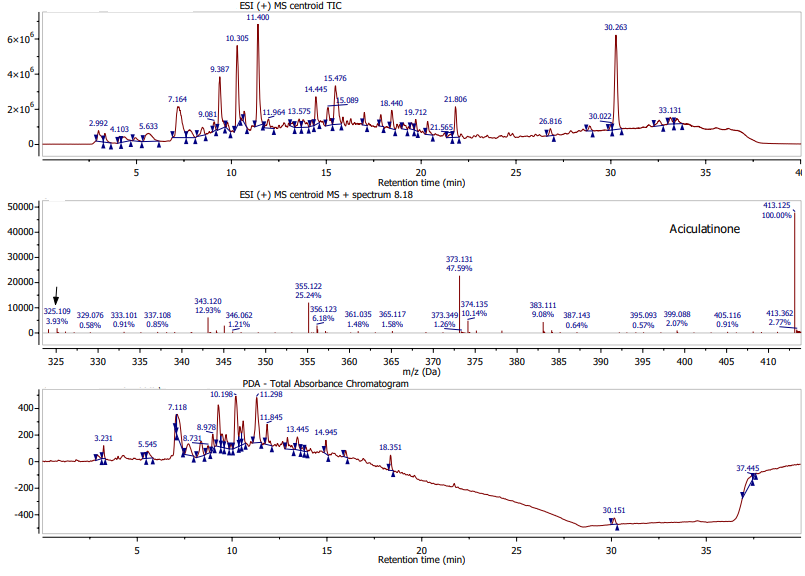


**Figure S21** Mass spectrum of aciculatinone


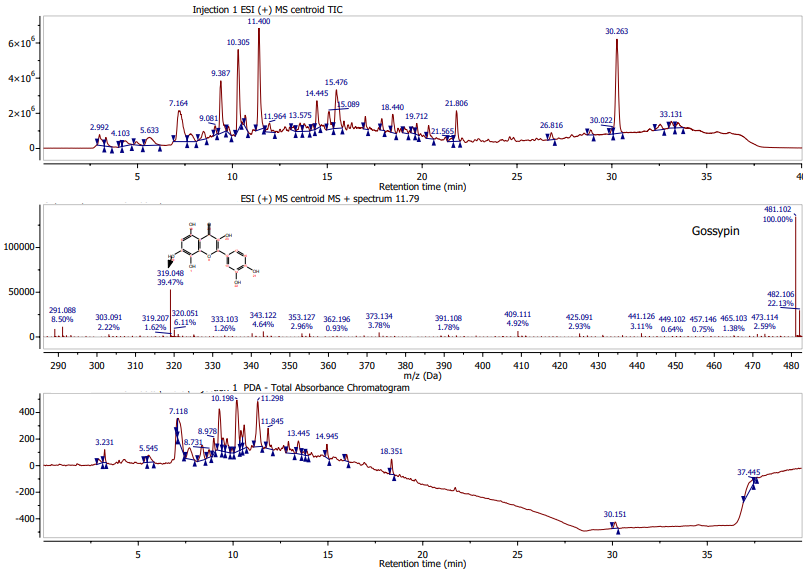


**Figure S22** Mass spectrum of gossypin


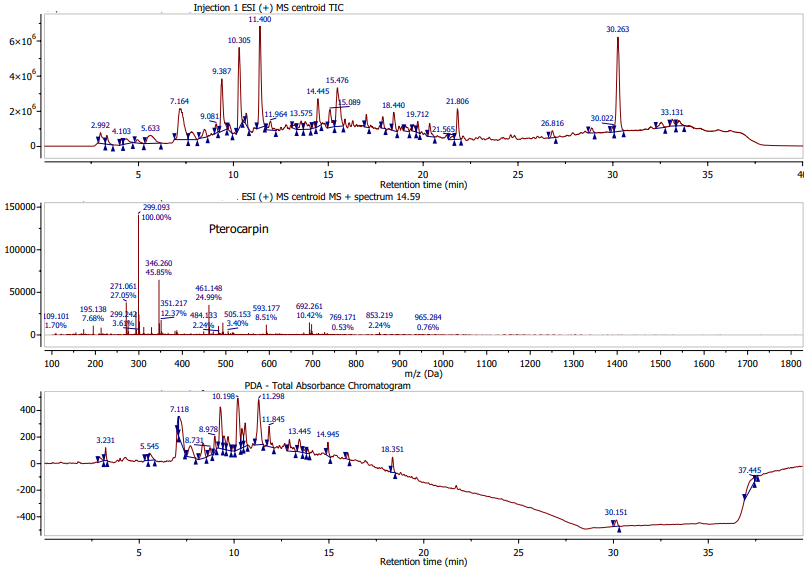


**Figure S23** Mass spectrum of pterocarpin


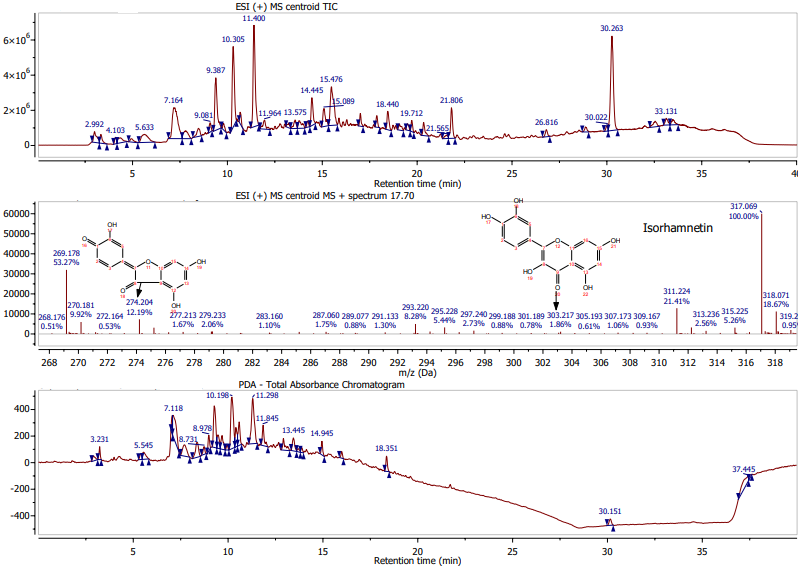


**Figure S24** Mass spectrum of isorhamnetin


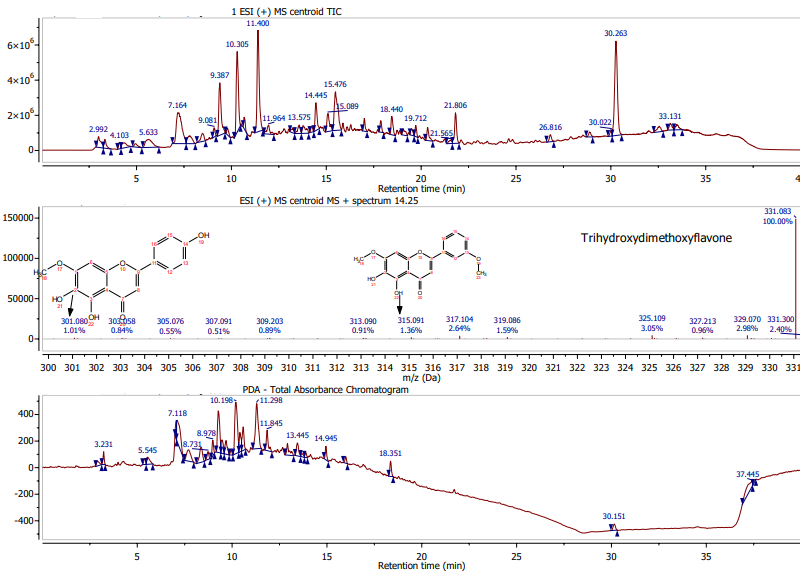


**Figure S25** Mass spectrum of trihydroxy dimethoxy flavone


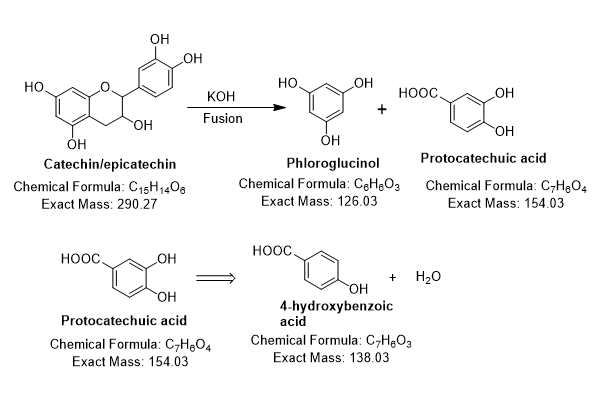


**Figure S26** Fragmentation pattern of catechin/epicatechin.


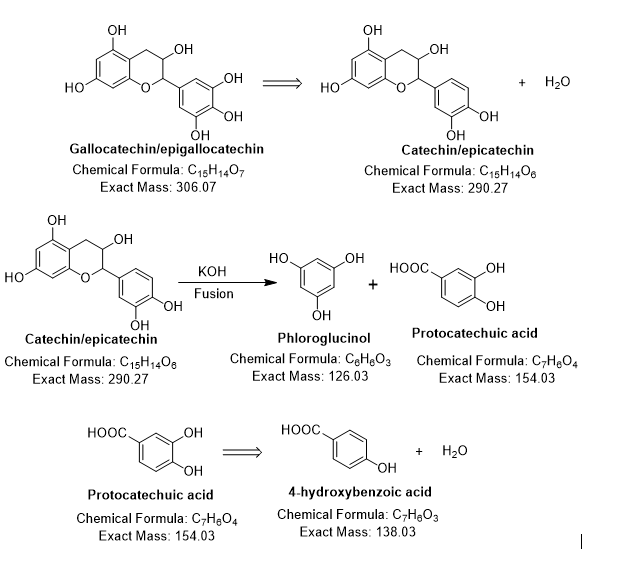


**Figure S27** Fragmentation pattern of gallocatechin/epigallocatechin


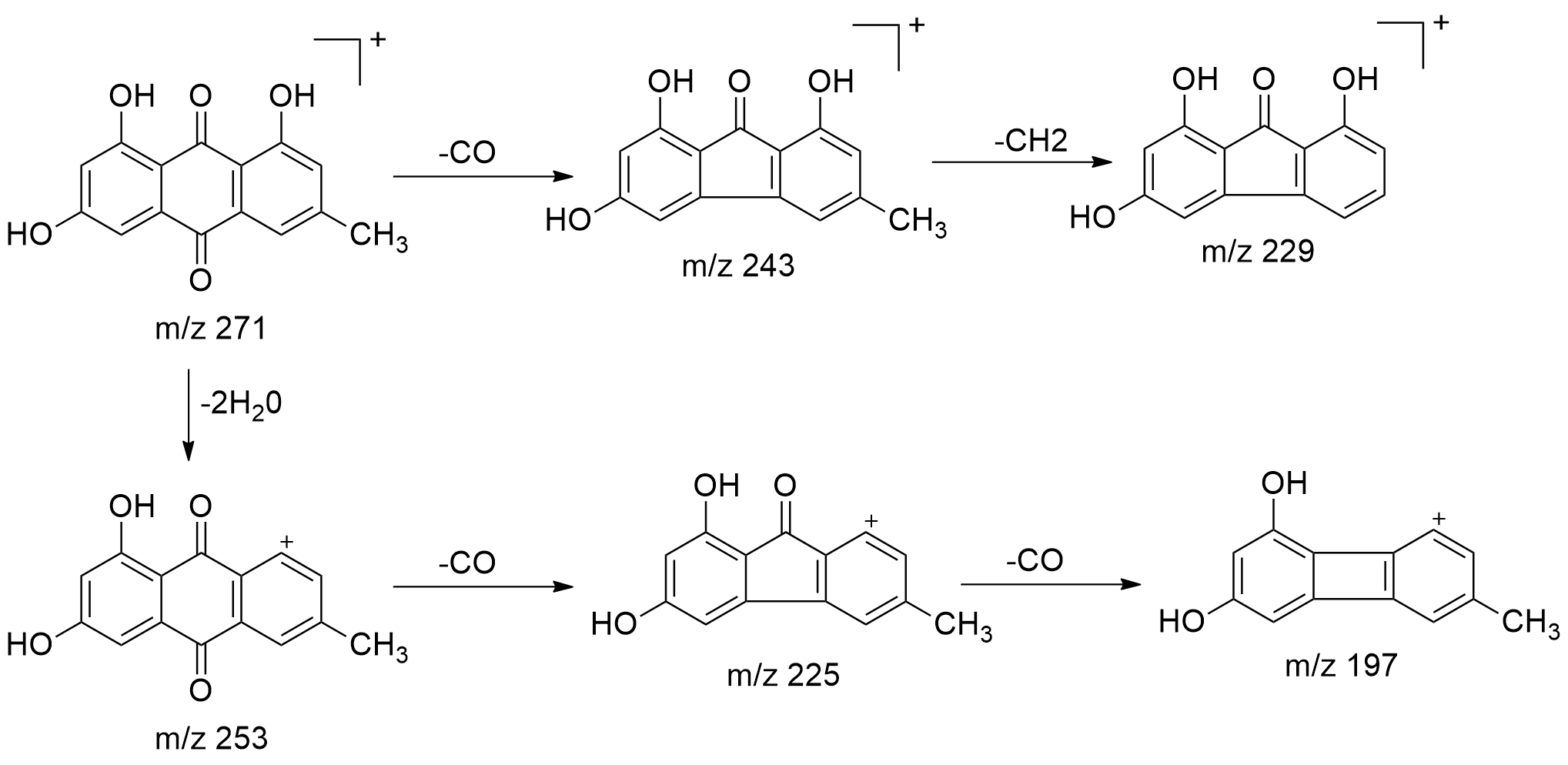


**Figure S28** Fragmentation pattern of emodin.

**Figure S29** Fragmentation pattern of naringenin

**Figure S30** Fragmentation pattern of isoquercetin

**Figure S31** Fragmentation pattern of diosmetin

**Figure S32** Fragmentation pattern of chrysin


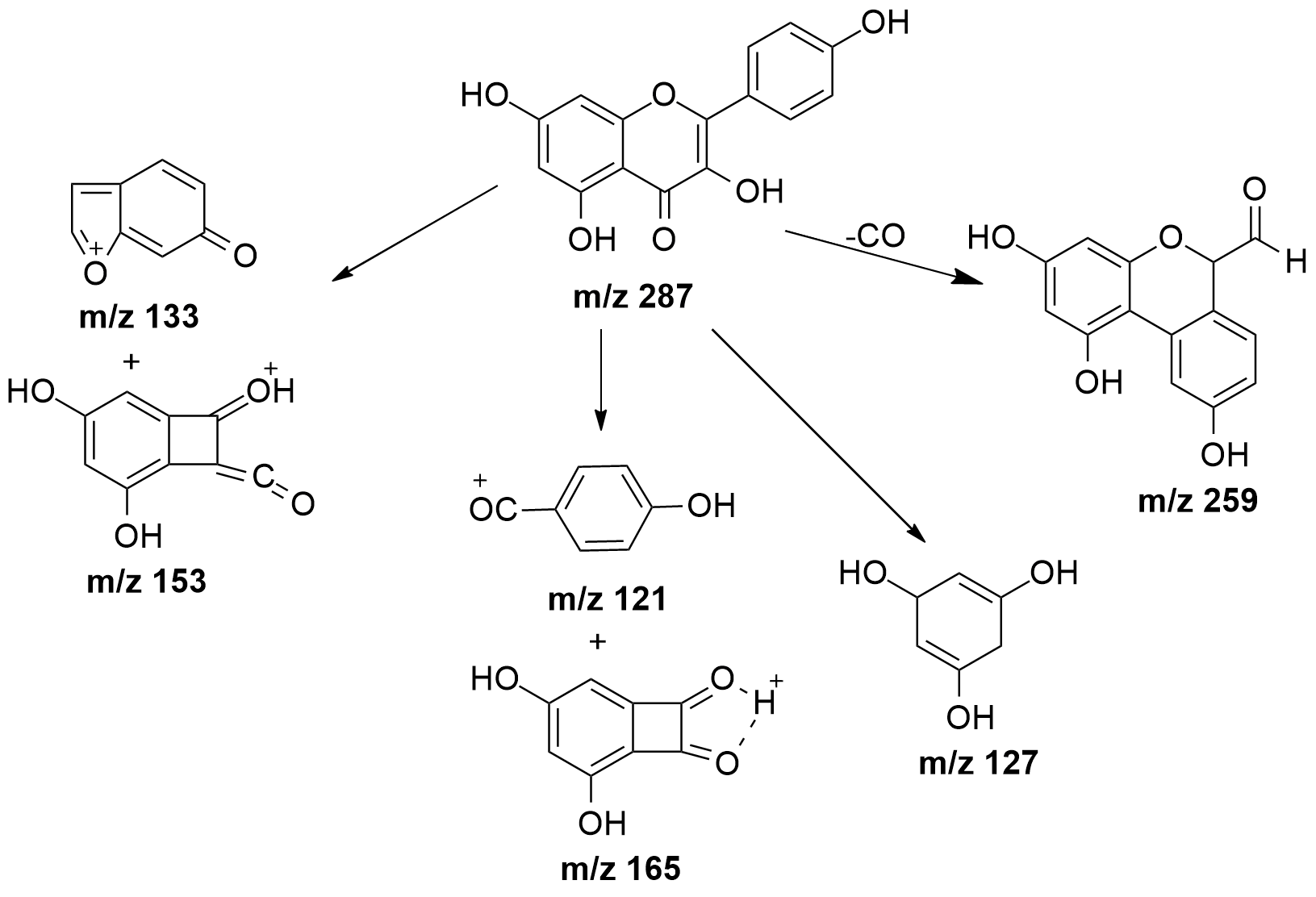


**Figure S33** Fragmentation pattern of kaempferol.

**Figure S34** Fragmentation pattern of avicularin

**Figure S35** Fragmentation pattern of prodelphinidin B3

**Figure S36** Fragmentation pattern of prodelphinidin B

**Figure S37** Fragmentation pattern of isorhamnetin

**Figure S38** Fragmentation pattern of trihydroxy-dimethoxyflavone
